# Biology-aware association for identity-consistent leaf tracking in top-down plant phenotyping

**DOI:** 10.3389/fpls.2026.1881413

**Published:** 2026-10-14

**Authors:** Dongze Zhou, Cong Zhang

**Affiliations:** 1 School of Mathematics and Computer Science, Wuhan Polytechnic University, Wuhan, Hubei, China; 2 School of Electrical and Electronic Engineering, Wuhan Polytechnic University, Wuhan, Hubei, China

**Keywords:** adaptive weighting, biology-aware association, *Brassica napus*, computer vision, leaf tracking, multi-object tracking, plant phenotyping, trait trajectory extraction

## Abstract

Reliable leaf-level trait trajectories require identity-consistent tracking across growth, overlap, and appearance change. We introduce a biology-aware association module combining one appearance cue and three biological/geometric cues: spatial continuity, area-growth plausibility, and lifecycle plausibility. On CanolaTrack, adaptive weighting reduces ID switches from 228 to 126 ± 5 (44.7%; per-plant paired *p* = 1.6 × 10^−5^, *n* = 37) and improves HOTA from 88.17 to 89.69 ± 0.10. Difficulty-stratified analysis shows IDSW reductions across all 12 CanolaTrack strata, averaging 40.0% versus Appearance-only and 45.0% versus LeafTrackNet. External association-only Arabidopsis and KOMATSUNA tests identify clean low-displacement GT-box regimes where centroid matching or Fixed bio-constraints are more reliable than zero-shot adaptive-weight transfer. These results provide an operating-envelope recommendation: adaptive weighting is most useful in dense CanolaTrack-like sequences, whereas Fixed bio-constraints or centroid matching are more reliable in clean low-displacement external regimes—where nearest-centroid assignment already resolves most associations, so that even a predictor retrained on the target domain does not surpass it. The limiting factor is thus primarily the association difficulty of the regime rather than dataset size.

## 1 Introduction

Automated plant phenotyping has become an important tool in modern crop science, enabling scalable, repeatable characterization of growth dynamics, stress responses, and developmental trajectories across large plant populations (Murphy et al., 2024; Tardieu et al., 2017). Among the morphological traits of interest, individual leaf-level measurements are particularly informative: leaves are the primary sites of photosynthesis, the earliest organs to display stress symptoms, and carriers of fine-grained temporal signals—including emergence timing, area growth rate, functional lifespan, and senescence onset—directly relevant to genotype evaluation and breeding program design (Cai et al., 2016; Yan et al., 2016; Poethig, 2013). Finely annotated plant image datasets (Minervini et al., 2016) and deep learning pipelines for stress detection (Jeong et al., 2025) have accelerated progress in this area, yet high-throughput systems capable of extracting leaf-level trait trajectories longitudinally at scale remain an open engineering challenge.

Maintaining consistent leaf identities across an extended image sequence—the task of leaf tracking—is a prerequisite for extracting these longitudinal phenotypic signals. In top-down imaging of rosette species, daily-interval acquisition introduces a set of challenges that are qualitatively different from standard video tracking. Consecutive observations are separated by 24-hour growth intervals during which leaves can change substantially in size, shape, and color. Occlusion in dense canopies is persistent: in the CanolaTrack benchmark (Liu et al., 2025b) for canola (*Brassica napus*) (Correndo et al., 2024), 38.5% of frames contain overlapping leaf pairs, no leaf remains visible throughout the full 31-day sequence, and 39.4% of consecutive-frame leaf pairs exhibit area changes exceeding 30%. Under these conditions, identity switches accumulate across time and corrupt downstream phenotypic trajectories: a single misassociation can corrupt subsequent measurements for one or both affected tracks.

General-purpose multi-object tracking (MOT) methods—including SORT (Bewley et al., 2016), DeepSORT (Wojke et al., 2017), FairMOT (Wang et al., 2021), ByteTrack (Zhang et al., 2022), OC-SORT (Cao et al., 2023), StrongSORT (Du et al., 2023), BoT-SORT (Aharon et al., 2022), and end-to-end approaches such as MOTRv2 (Zhang et al., 2023)—are developed primarily on driving and human-tracking benchmarks such as BDD100K (Yu et al., 2020) and DanceTrack (Sun et al., 2022). Many of these methods rely on Kalman-filter motion prediction, IoU-based matching, or high-frame-rate temporal continuity assumptions, often with near-rigid object appearance. These assumptions are violated in daily-interval plant imaging, where 24-hour intervals render predicted positions unreliable and leaves grow significantly between observations. Plant-specific methods represent a step forward: LeTra (Jurado-Ruiz et al., 2024) employs Mask R-CNN (He et al., 2017) for instance segmentation with IoU-based matching; PlantDoctor (Montagut-Marquès et al., 2025) integrates deep detection with appearance-based tracking for plant damage quantification; Weyler et al. (2021) address joint leaf detection and counting but without maintaining persistent cross-frame identities; and LeafTrackNet (Liu et al., 2025b) combines a YOLOv10 (Wang et al., 2024) detector with MobileNetV3 (Howard et al., 2019) appearance embeddings and memory-based Hungarian assignment, achieving substantially better performance on CanolaTrack. However, these methods either rely on appearance similarity as the primary matching signal or use simple geometric overlap, and none systematically combines multiple complementary association cues or tests how association rules behave across different plant growth and ambiguity regimes.

Biological and geometric cues offer a complementary source of information for association. During active vegetative growth, leaf lamina area tends to increase over time; leaf centroids usually move slowly relative to image dimensions; and briefly occluded leaves often reappear close to their last known positions (Poethig, 2013; Granier et al., 2002). These properties translate naturally into soft constraints on the association cost matrix. At the same time, the most reliable association strategy may depend on operating regime: dense canopies and overlapping leaves require stronger biological and geometric checks, whereas small clean datasets with minimal displacement may be well served by simple centroid matching. A comprehensive evaluation therefore needs to measure both average benchmark accuracy and the deployment boundary of each association strategy.

This paper addresses that gap by presenting a biology-aware association module designed as a drop-in replacement for the matching stage of compatible tracking-by-detection pipelines for top-down plant phenotyping. The study also evaluates the operating envelope of adaptive and fixed association rules using CanolaTrack difficulty strata, external GT-box association-only tests, proposal perturbations, and diagnostic analysis. The specific contributions are:

1. A biology-aware association framework that combines one appearance cue with three biological/geometric cues—spatial continuity, area-growth plausibility, and lifecycle plausibility—in a unified Hungarian assignment cost for daily-interval leaf tracking. We instantiate the framework in two variants: a *fixed-weight* variant (our non-learned biology-aware association) and an *adaptive* per-pair weight-predictor variant (our learned extension).
2. A contribution decomposition on CanolaTrack: the fixed biology-aware constraints recover most of the identity-switch reduction over the prior baseline (228 to 147 IDSW; per-plant *p* = 1.3 × 10^−4^), and the adaptive Weight Predictor (∼2,800 parameters; pairwise ranking loss (Burges et al., 2005)) adds a further modest, targeted reduction (147 to 126 IDSW; nominally significant at *p* = 0.049) that is concentrated in spatially ambiguous sequences.
3. A difficulty-stratified and trajectory-reliability analysis showing that the adaptive benefit is *targeted*— largest under high spatial ambiguity—rather than a uniform improvement, and that biology-aware association yields more reliable per-leaf trajectories.
4. An external operating-envelope analysis (association-only, with target-domain calibration) that establishes deployment boundaries: adaptive weighting suits dense CanolaTrack-like sequences, while centroid or fixed biology-aware constraints are more reliable in clean, low-displacement regimes. These external tests define an operating boundary and are not a detector-transfer or universal-generalisation claim.

## 2 Materials and methods

### 2.1 Dataset: CanolaTrack

The primary experiments used the CanolaTrack dataset (Liu et al., 2025a), following the benchmark protocol described by Liu et al. (2025b); the external Arabidopsis and KOMATSUNA evaluations are described separately in Section 2.5. The dataset is a publicly available benchmark for top-down leaf tracking comprising 5,704 RGB images (1200 × 1200 pixels) of 184 *Brassica napus* (canola) plants acquired at daily intervals over 31 consecutive days, with 31,840 annotated leaf instances carrying persistent identity labels. The dataset was partitioned into 147 training plants and 37 validation plants (1,147 images, 6,355 annotated leaf instances). Official pre-computed detection proposals and appearance embeddings from the LeafTrackNet pipeline described by Liu et al. (2025b) and released in its official repository (Liu et al., 2025c) were used throughout all CanolaTrack experiments to ensure a strictly comparable evaluation.

**Table 1** summarizes the validation-set statistics, and **Figure 1** illustrates the tracking challenges, that motivate the design of the four constraint terms. Three characteristics are particularly relevant. First, the mean leaf lifespan is only 12.46 ± 6.62 frames and no leaf remains visible for the full 31-day sequence, confirming that entries and exits of leaf tracks are frequent events requiring robust handling. Second, the mean occlusion rate is 38.5 ± 10.0%, creating systematic ambiguity for appearance-only matchers. Third, 39.4% of consecutive-frame leaf pairs exhibit area changes exceeding 30%, while the median centroid displacement between frames is only 22.69px relative to the 1200 × 1200 image—motivating a soft area growth prior alongside a spatial continuity constraint.

**Table 1:** CanolaTrack validation set statistics (37 plants, 1,147 frames, 6,355 leaf instances).

Statistic	Value
Plants/Frames/Leaf instances	37/1,147/6,355
Mean leaf count per frame	5.54 ± 2.60
Mean unique leaf IDs per plant	13.78 ± 1.02
Mean leaf lifespan (frames)	12.46 ± 6.62
Leaves visible all 31 days	0%
Median |Δ*A/A*| per frame	0.218
Pairs with >30% area change	39.4%
Median centroid displacement (px)	22.69
Mean occlusion rate (IoU*>*0.1)	38.5 ± 10.0%

Occlusion rate is the fraction of frames containing any leaf pair with IoU*>*0.1, averaged over plants; |Δ*A/A*| and centroid displacement are computed per same-identity consecutive-frame leaf pair; the >30% area-change figure is the fraction of such pairs exceeding that threshold.

**Figure 1:**
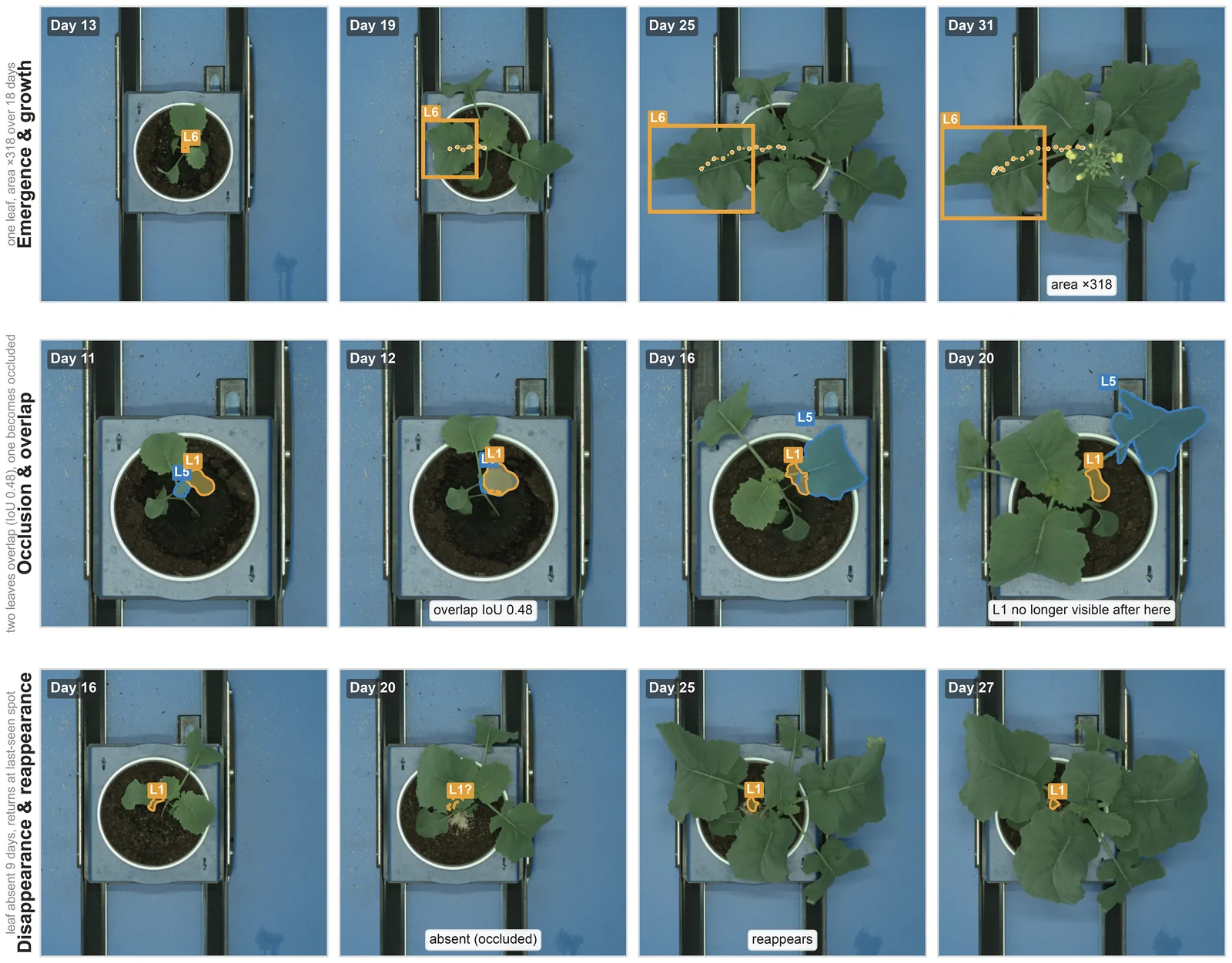
Three tracking challenges on CanolaTrack, each illustrated by following one or two selected ground-truth leaves across days. All three panels show the same species (*Brassica napus*); the three plants are the validation individuals that most clearly exhibit each challenge under an objective criterion. Top—emergence and growth (largest single-leaf area growth in the validation set): leaf L6 grows ∼318× in area from Day 13 to Day 31, with its centroid trajectory marked by the dotted trail. Middle—occlusion and overlap (largest inter-leaf overlap): leaves L1 and L5 overlap (IoU 0.48 at Day 12) and L1 is no longer visible or annotated after Day 20. Bottom—disappearance and reappearance (longest absence gap): leaf L1 is last clearly visible at Day 16, is absent (occluded) at Day 20, and reappears at Day 25—nine days after its last observation—at almost the same location. Coloured overlays highlight the tracked leaf in each panel; the bounding boxes and leaf identities are the ground-truth tracking annotations used throughout.

### 2.2 Tracking-by-detection baseline

The LeafTrackNet pipeline (Liu et al., 2025b) serves as the direct baseline and provides the shared detection and embedding components used across the CanolaTrack comparisons. A YOLOv10 (Wang et al., 2024) detector produces per-frame leaf bounding box proposals; a MobileNetV3 (Howard et al., 2019) encoder pre-trained on ImageNet (Deng et al., 2009) and fine-tuned with triplet loss (Balntas et al., 2016) extracts 128-dimensional appearance embeddings for each proposal. At each time step, a memory bank stores *M* active tracks, each maintaining a prototype embedding $p^{l}\in {\mathbb{R}}^{128}$, centroid $c^{l}$, area estimate $A^{l}$, and an age counter $a^{l}$ recording frames since last successful association. Matching is performed by cosine-distance nearest assignment under Hungarian optimisation (Kuhn, 1955). The proposed method modifies only the association cost matrix; the detector and encoder remain unchanged. An overview of the complete biology-aware tracking-by-detection framework is shown in **Figure 2**. For all CanolaTrack comparisons, the evaluated methods use the same set of pre-computed detection proposals and, where appearance is used, the same LeafTrackNet embeddings, isolating the contribution of the association strategy from any differences in detection or feature-extraction quality. (The external experiments instead use GT-derived boxes/masks as proposals and zero-shot ImageNet-pretrained MobileNetV3 crop embeddings, as detailed in the external protocol).

**Figure 2:**
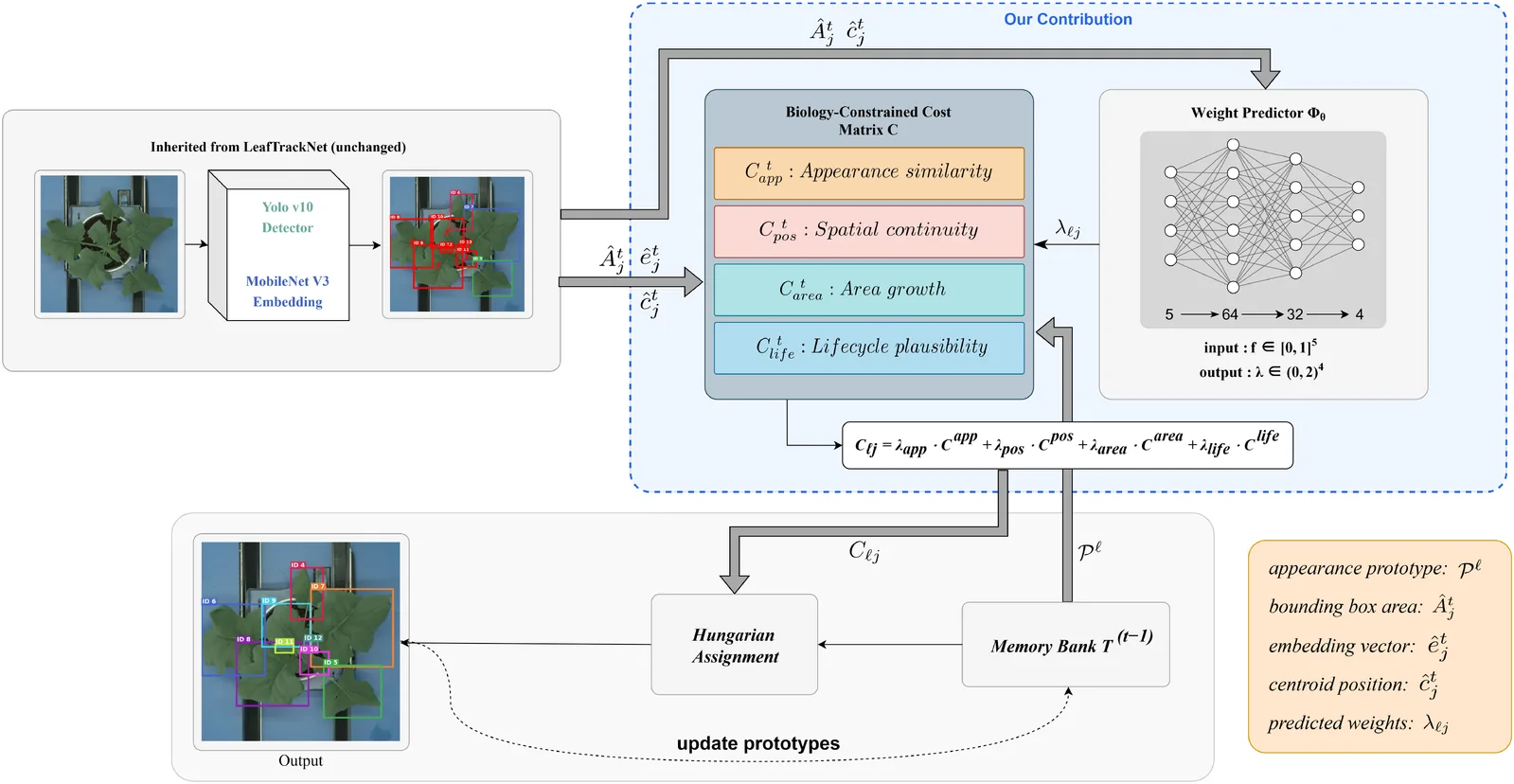
Overview of the biology-aware tracking-by-detection framework. Grey components (YOLOv10 detector, MobileNetV3 encoder, memory bank) are inherited unchanged from LeafTrackNet (Liu et al., 2025b). The blue dashed region highlights the proposed contribution: a biology-constrained cost matrix comprising one appearance cue and three biological/geometric cues, and an adaptive Weight Predictor Φ*_θ_*that infers per-pair constraint weights from geometric features.

### 2.3 Biology-constrained cost function

Let $D^{t}={\left\{{\hat{b}}_{j}^{t}\right\}}_{j=1}^{N_{t}}$ denote the set of *N_t_* detection proposals at time *t*, and let T *^t^*^−1^ be the memory bank of *M* active tracks. The goal is a minimum-cost one-to-one *partial* matching *π*^∗^ between active tracks and current detections that preserves leaf identities; unmatched tracks and detections are permitted and are handled by the track-management rules below. The composite association cost for track *ℓ* and detection *j* is:

(1) $$C_{lj}=\lambda_{\text{app}}^{lj}\;C_{lj}^{\text{app}}+\lambda_{\text{pos}}^{lj}\;C_{lj}^{\text{pos}}+\lambda_{\text{area}}^{lj}\;C_{lj}^{\text{area}}+\lambda_{\text{life}}^{lj}\;C_{lj}^{\text{life}},$$

where the appearance cost and three biological/geometric cost terms, together with their associated weights, are described below. Each term targets a specific challenge identified in Section 1: the appearance cost addresses growth- and illumination-induced changes in leaf appearance; the spatial continuity cost addresses inter-leaf overlap and centroid ambiguity; the area-growth cost addresses daily lamina size change; and the lifecycle cost addresses leaf disappearance and reappearance.

#### 2.3.1 Appearance cost

Standard cosine dissimilarity between the track prototype embedding and the detection embedding:

(2) $$C_{\ell j}^{\text{app}}=1-\frac{p^{\ell }\cdot {\hat{e}}_{j}^{t}}{\left\|p^{\ell }\right\|\left\|{\hat{e}}_{j}^{t}\right\|},$$

where ${\hat{e}}_{j}^{t}\in {\mathbb{R}}^{128}$ is extracted by the frozen MobileNetV3 encoder.

#### 2.3.2 Spatial continuity cost

During the vegetative phase, leaf centroids move slowly relative to image dimensions. Large inter-frame displacements are therefore biologically less plausible and penalised as: .

(3) $$C_{lj}^{\text{pos}}=\text{min }(\frac{\|c^{l}-{\hat{c}}_{j}^{t}{\|}_{2}}{d_{\text{max}}\cdot r},\;1),$$

where *r* is the image width and *d*_max_ = 0.25 is the maximum normalized spatial distance. Displacements exceeding *d*_max_ · *r* pixels are clamped to 1.

#### 2.3.3 Area growth cost

Leaf lamina area tends to increase during active vegetative growth; abrupt apparent shrinkage between consecutive daily observations is treated as a biologically less plausible association (Poethig, 2013; Granier et al., 2002). This is encoded as a soft, one-sided area-shrinkage plausibility prior that penalises candidate matches implying substantial area decrease while remaining permissive of moderate fluctuations arising from detection noise or partial occlusion, and imposing no penalty on area increase:

(4) $$C_{lj}^{\text{area}}=\text{min}(\text{max}(0,\;\frac{A^{l}-{\hat{A}}_{j}^{t}}{A^{l}}-\tau_{A})\cdot s_{A},\;1),$$

with tolerance *τ_A_*= 0.30 and penalty scale *s_A_*= 1.0. Matches implying no more than 30% area shrinkage incur zero cost; area increases, of any magnitude, are not penalised by this one-sided prior.

Both the spatial and area terms are deliberately soft: each is a capped, tolerance-based penalty rather than a hard gate. Occasional large centroid displacements or apparent area changes—for example under pot rotation—therefore raise but do not veto an association, and can still be outweighed by a strong appearance match. The adaptive Weight Predictor (Section 2.4) provides a further safeguard by downweighting the spatial and area terms for pairs whose geometric features indicate that these cues are unreliable.

#### 2.3.4 Lifecycle plausibility cost

A track that has been absent for several frames is increasingly unlikely to reappear at a location far from its last known centroid. This plausibility decreases with track age $a^{l}$:

(5) $$C_{lj}^{\text{life}}=\text{min }(\frac{a^{l}}{\tau_{a}},\;1)\cdot \text{min }(\frac{\|c^{l}-{\hat{c}}_{j}^{t}{\|}_{2}}{d_{\text{max}}\cdot r},\;1),$$

where *τ_a_*= 5 is the track-age scale (in frames), and both the age factor and spatial factor are clamped to 1. A track absent for many frames therefore incurs higher cost for associations with spatially distant detections. By construction this term is an age-gated form of the spatial cost: it is inactive for currently visible tracks ( $a^{l}=0$) and contributes only when re-associating tracks that have been absent for one or more frames. It is therefore a targeted prior for the leaf disappearance and reappearance challenge rather than a cue that acts on every association.

For the Fixed bio-constraints configuration, we use the full fixed-weight version of the association cost, including appearance similarity, spatial continuity, area-growth plausibility, and lifecycle plausibility. The weights are set to $\lambda_{\text{app}}=1.0$, $\lambda_{\text{pos}}=0.3$, $\lambda_{\text{area}}=0.2$, and $\lambda_{\text{life}}=0.1$. The optimal assignment is obtained via the Hungarian algorithm (Kuhn, 1955) on this composite cost; an assigned pair is then accepted only if its appearance cosine similarity is at least $\tau_{\text{app}}=0.4$, and pairs below this threshold are rejected. Unmatched tracks are retained in the memory bank for up to $\tau_{a}=5$ frames before removal.

All constraint parameters were selected on the 147-plant training split and held fixed for all primary CanolaTrack benchmark comparisons (the designated sensitivity sweeps of **Supplementary Table S1** deliberately vary them one at a time); the 37-plant validation set was not used for any parameter tuning. The spatial scale *d*_max_ = 0.25, area tolerance *τ_A_*= 0.30, penalty scale *s_A_*= 1.0, and age scale *τ_a_*= 5 instantiate the biological priors motivated in Section 2.1 (slow centroid motion, growth-dominated area change, and short reappearance windows). These are heuristic settings read off training-plant statistics and these priors—for example, *τ_A_*and *τ_a_*reflect the typical per-interval area change and reappearance gap on the training plants—rather than the result of a formal search on any held-out objective; the sensitivity analysis (**Supplementary Table S1**) confirms that the results are robust to them rather than being used to select them. The blend weights (*λ*_app_=1.0, *λ*_pos_=0.3, *λ*_area_=0.2, *λ*_life_=0.1) and the appearance-cosine rejection threshold *τ*_app_ = 0.4 were likewise set from training-split behavior and reused unchanged across the primary CanolaTrack benchmark and ablation comparisons, except for the explicitly designated sensitivity sweeps (**Supplementary Table S1**). On the external datasets the default rows adopt the same numerical settings, except that the acceptance test there is applied to the composite association cost with threshold *τ_c_*= 0.4 (a distinct quantity on the cost scale; see Section 3.3). The target-domain *calibrated* variants used only in the external evaluation instead re-select these on a held-out target-domain calibration split, never on test data, as detailed there. A one-at-a-time sensitivity analysis of the principal association hyperparameters (**Supplementary Table S1**) confirms that these values are not highly sensitive to a narrow parameter range: across all tested configurations HOTA remains within a 0.66-point band ([88.47, 89.13]) and IDSW within [143, 161], with the default on a broad near-optimal plateau and the largest single effect coming from loosening the spatial normalisation scale *d*_max_.

Track management follows the memory-bank baseline. Assignment is solved once per frame by the Hungarian algorithm on the composite cost, after which an assigned pair is accepted only if its appearance cosine similarity is at least *τ*_app_; assignments below *τ*_app_ are rejected (i.e. rejection is applied *after* the Hungarian solve). After each accepted assignment, a matched track updates its prototype embedding to the running arithmetic mean of all its matched detection embeddings (the summed embeddings divided by the match count; used for all reported results), which is *L*_2_-normalised before scoring; it refreshes its stored centroid and area to those of the most recent matched detection, and resets its age counter to zero. Each detection embedding is itself *L*_2_-normalised. Each unmatched detection initialises a new track with a fresh identity; each unmatched track increments its age and is retained as a coasting track—remaining eligible for association—until its age exceeds *τ_a_*= 5 frames, after which it is pruned from the memory bank.

### 2.4 Adaptive Weight Predictor

Fixed weights treat all track–detection pairs uniformly, but the relative discriminative power of each constraint depends on the biological state of the leaf pair. We hypothesise, for example, that a newly emerged small leaf may be better matched by upweighting appearance and downweighting the area constraint, or that a briefly occluded leaf may be better matched by relying more on spatial continuity; the learned weight distributions that bear on these hypotheses are reported in Section 3.5. As detailed below, the training pairs are gap-free consecutive-frame transitions, so the lifecycle branch is not supervised and we do not attribute any learned lifecycle-specific behavior to the predictor. To capture this context dependency, this study introduces an adaptive Weight Predictor Φ*_θ_* that maps geometric features to per-pair constraint weights.

#### 2.4.1 Geometric feature vector

For each track–detection pair (*ℓ,j*), a five-dimensional feature vector is computed from observable geometric quantities:

(6) $$f_{\ell j}=\left[\frac{\begin{array}{c} \min\left(\frac{{\hat{A}}_{j}^{t}}{4\;A^{\ell }},\;1\right)\\ 1+\text{clip}\left(\frac{{\hat{A}}_{j}^{t}-A^{\ell }}{A^{\ell }},\;-1,\;1\right) \end{array}}{\begin{array}{c} 2\\ \begin{array}{c} \min\left(\frac{{\left\|c^{\ell }-{\hat{c}}_{j}^{t}\right\|}_{2}}{r},\;1\right)\\ \begin{array}{c} \min\left(\frac{a^{\ell }}{\tau_{a}},\;1\right)\\ \frac{t}{T} \end{array} \end{array} \end{array}}\right]\in {\left[0,1\right]}^{5},$$

encoding relative area ratio, normalized area change, normalized spatial displacement, normalized track age, and fractional time position within the sequence, respectively. The area-ratio component is divided by four and clipped to [0,1] so that it saturates when a candidate detection reaches four times the track area; this bounds the feature to the same [0,1] range as the other components while spanning the plausible per-interval growth range. The fractional time position *t/T* assumes a known sequence length; all CanolaTrack sequences share *T* = 31, so this component is well defined here, whereas variable-length deployments would require a length estimate or a bounded elapsed-time proxy. The marginal contribution of each feature is quantified by a leave-one-out ablation (**Supplementary Table S3**), in which each component is masked identically at training and inference and the predictor retrained: the area-ratio and normalized-displacement components carry most of the association benefit (removing them raises IDSW by 15 and 10 respectively). Removing the track-age feature *f*_3_ leaves IDSW unchanged (127), because *f*_3_ is inactive under the present gap-free training construction (the age is identically zero during training; Section 2.4) rather than merely redundant; the relative-area-change and fractional sequence-position features contribute marginally. The full five-feature model therefore ties for the lowest IDSW and degrades gracefully under any single-feature removal. Equations 1–7 define the composite association cost, its component costs, the geometric feature vector, and the adaptive per-pair weighting used by the proposed method.

#### 2.4.2 Architecture and training

Φ*_θ_* is a three-layer MLP with architecture 5 → 64 → 32 → 4, using LayerNorm (Ba et al., 2016) and ReLU activations. The output is passed through a sigmoid and scaled by *λ*_max_ = 2.0 to produce non-negative per-pair weights:

(7) $$\lambda_{lj}=\sigma ({\text{Φ}}_{\theta }(f_{lj}))\cdot \lambda_{\text{max}}.$$

Training pairs were constructed directly from the ground-truth MOT annotations of the 147 training plants; no model predictions were used to form the training labels. We fully enumerate the eligible positive transitions: for every consecutive frame pair (*t,t* + 1) in a plant sequence, each leaf present at *t* that persists to *t* + 1 (a same-identity detection exists) and co-occurs with at least one different-identity detection at *t* + 1 contributes one anchor–positive pair. This enumeration yields exactly 23,234 eligible positives across the 147 plants (verified programmatically; per-plant counts recorded in the accompanying log). For each positive, a single negative is drawn uniformly from the same-frame different-identity detections *once at dataset construction* and then held fixed—negatives are not resampled per epoch. No explicit hard-negative mining is applied; same-frame different-identity leaves provide plausible competing negatives for association. Each training epoch draws 20,000 triplets with replacement from this fixed pool; the random seed jointly controls the negative selection, the data-loading order, and the model initialisation, so the three seeds (42/123/456) train on different negative sets. The objective is a pairwise ranking loss (Burges et al., 2005), conceptually related to metric-learning objectives used in re-identification (Schroff et al., 2015; Balntas et al., 2016), which encourages the weighted composite cost to be lower for ground-truth positive pairs than for negative pairs within the same frame:

(8) $$ℒ=\frac{1}{\left|ℬ\right|}\;\sum_{\left(\ell ,\;j^{+},\;j^{-}\right)\in ℬ}\text{ max }\left(0,\;\lambda_{\ell j^{+}}\cdot c_{\ell j^{+}}-\lambda_{\ell j^{-}}\cdot c_{\ell j^{-}}+m\right),$$

where $c_{lj}={\left[C_{lj}^{\text{app}},\;C_{lj}^{\text{pos}},\;C_{lj}^{\text{area}},\;C_{lj}^{\text{life}}\right]}^{⊤}$ is the ground-truth match for track $l,\;j^{-}$ is a negative detection sampled from the same frame, and *m* = 0.1 is the ranking margin. During training, $C^{\text{app}}$ is replaced by a proxy value fixed at 0.5 to prevent the predictor from overfitting to the appearance signal.

Two properties of this construction constrain what the predictor can learn about the lifecycle term, and we state them explicitly. The track state feeding each pair is taken from the frame-*t* ground-truth box (the anchor centroid and area are those of the leaf at *t*), and because every training pair is a gap-free consecutive-frame transition, the track age is fixed to 0 for all pairs. The age feature *f*_3_ is therefore identically zero during training, and the age-gated lifecycle cost $C^{\text{life}}$ is identically zero for both positive and negative pairs. Missing detections and leaf reappearance are not simulated. Consequently the lifecycle output weight *λ*_3_ multiplies a constant-zero cost in the ranking loss (Equation 8) and receives no direct gradient signal. Because $C^{\text{app}}$ is fixed at the constant proxy, the objective carries no discriminative appearance-*embedding* information; however, the appearance-*weight* output can still receive gradient, since the predicted $\lambda^{\text{app}}$ is pair-dependent (its positive- and negative-pair values differ) and enters the loss as $0.5(\lambda_{+}^{\text{app}}-\lambda_{-}^{\text{app}})$. The predictor can therefore learn a geometry-conditioned modulation of the appearance weight, while the discriminative geometric signal comes from the position and area costs (which differ between positive and negative pairs). We therefore do not claim that the predictor learns any lifecycle-specific or reappearance-specific weighting; the lifecycle output branch is unsupervised under this construction, and gap-aware lifecycle learning is left to future work (Section 4.5).

The model was optimized with Adam (Kingma and Ba, 2015) at learning rate 10^−3^ with weight decay 10^−5^, cosine annealing (Loshchilov and Hutter, 2017) over 100 epochs, batch size 512, and gradient clipping at 1.0. Three independent training runs with seeds 42, 123, and 456 were conducted; the primary adaptive benchmark results are reported as mean ± std over these three seeds, and the supplementary ablations state their own seed protocol separately. The per-seed tracking metrics are reported in **Supplementary Table S2**: they are tightly clustered (HOTA standard deviation ±0.10) and every seed outperforms the fixed-weight baseline on HOTA, AssA, IDF1, MOTA, and IDSW, with DetA effectively unchanged under the shared detection proposals, confirming that the adaptive improvement is reproducible across initialisations rather than an artefact of a single run.

### 2.5 Evaluation protocol

#### 2.5.1 Tracking metrics

Standard MOT metrics were computed via TrackEval (Luiten et al., 2021): Higher Order Tracking Accuracy (HOTA), Detection Accuracy (DetA), Association Accuracy (AssA), Multi-Object Tracking Accuracy (MOTA), Identification F1 (IDF1), and Identity Switches (IDSW). For the CanolaTrack evaluation, all methods used the same pre-computed detection proposals; appearance-based methods additionally used the same embedding features. Because LeafTrackNet and the proposed variants share identical detection proposals and output all detections without additional filtering, their DetA is nearly identical (LeafTrackNet 92.25, Ours 92.27), differing only within run-to-run variation. For adapted general-purpose trackers, DetA differs because each tracker applies its own track initiation, confirmation, and termination logic, which may suppress detections that do not meet minimum confidence or track-length thresholds.

#### 2.5.2 Downstream phenotyping metrics

To evaluate the impact of tracking accuracy on phenotypic measurements, predicted tracks were matched to ground-truth leaf identities using temporal intersection-over-union (tIoU) via Hungarian assignment, with a minimum tIoU threshold of 0.3. Each ground-truth leaf was matched to at most one predicted track; unmatched ground-truth leaves were excluded from per-leaf emergence, lifespan, and area-correlation error computation but were reported separately as matching statistics. Four trajectory-derived metrics were computed: (1) emergence day MAE, the mean absolute error between predicted and annotated first-appearance frame, computed per plant; (2) lifespan MAE, the mean absolute error in visible lifespan length, computed per plant; (3) leaf count RMSE, the root-mean-square error of the number of active leaf identities per frame, averaged per plant; and (4) area trajectory correlation, the Pearson correlation between predicted and annotated per-leaf bounding-box area trajectories for matched leaves, averaged per plant. Area correlations were computed over co-observed frames and required at least three common frames. All metrics are reported as per-plant means across the 37 validation plants.

#### 2.5.3 Statistical testing

Two-sided Wilcoxon signed-rank tests (Wilcoxon, 1945) were performed on the 37 validation plants under a paired design to compare the proposed method against LeafTrackNet (and against Fixed bio-constraints). For the adaptive method, the per-plant value entering the *tracking-metric* paired analyses—the per-plant IDSW improvement figure (**Figure 3**), the difficulty-stratified analysis, and the tracking-metric Wilcoxon tests—is the per-plant mean over the three Weight Predictor seeds (42/123/456); the deterministic baselines use their single per-plant value. The downstream leaf-level phenotyping metrics (**Table 2**), their paired Wilcoxon tests (**Table 3**), and the associated matched-leaf counts are instead computed on the adaptive tracks from seed 42, so that each plant contributes one internally consistent set of leaf trajectories to the trait-error analysis rather than a cross-seed average of per-leaf values. *p*-values are computed with scipy.stats.wilcoxon on the paired per-plant differences. For the primary per-plant IDSW comparisons (**Figure 3**), effect sizes are additionally summarised by the median paired difference with a 95% percentile bootstrap confidence interval (10,000 resamples of the 37 paired differences with replacement, fixed seed); the downstream phenotyping comparisons report the mean difference and Wilcoxon *p*-value only. Uncorrected two-sided *p*-values are reported in **Table 3**. Bonferroni correction over the four downstream phenotyping metrics was used for multiplicity-aware interpretation, with an adjusted threshold of *α* = 0.0125. Significance symbols in **Table 3** denote uncorrected two-sided *p*-values.

**Figure 3:**
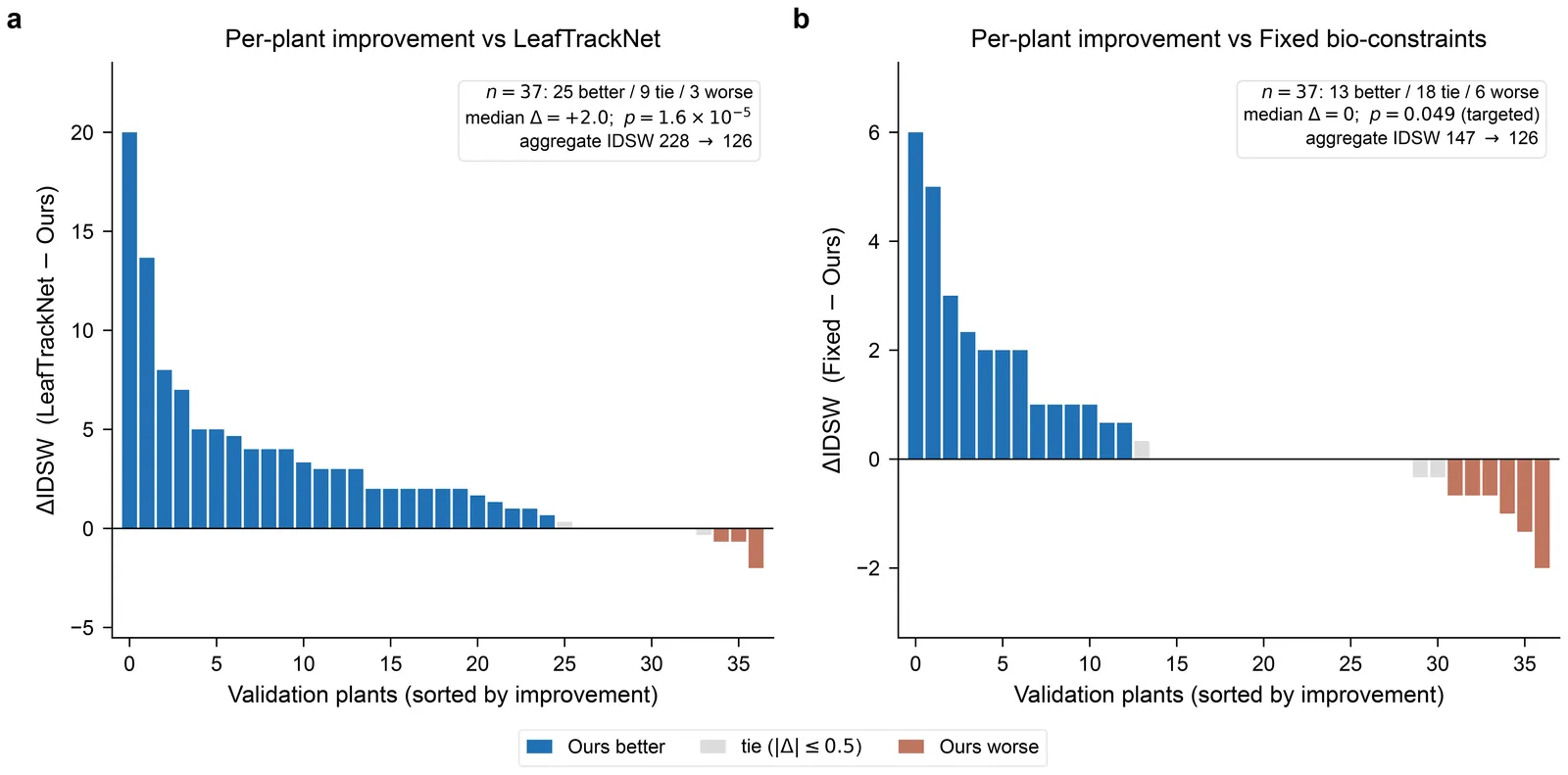
Per-plant paired improvement in identity switches on the 37 CanolaTrack validation plants (complements the aggregate totals in **Table 5**). Each bar is one plant, sorted by improvement and coloured by outcome; ΔIDSW *>* 0 denotes fewer identity switches for Ours. **(A)** vs the prior LeafTrackNet baseline: 25 plants improve, 9 tie, 3 worsen (two-sided per-plant Wilcoxon *p* = 1.6×10^−5^). **(B)** vs Fixed bio-constraints: 13 improve, 18 tie, 6 worsen (*p* = 0.049). Median differences, bootstrap intervals, and aggregate IDSW totals are given in Section 4.1 and **Table 5**.

**Table 2:** Downstream phenotyping accuracy on the 37-plant validation set.

Method	Emergence MAE↓	Lifespan MAE↓	LeafCount RMSE↓	Area Corr↑	Matched leaves↑
LeafTrackNet (Liu et al., 2025b)	0.191	0.505	0.461	0.926	504
Fixed bio-constraints	0.533	1.422	0.395	0.799	465
**Ours (Adaptive)**	**0.155**	**0.391**	**0.367**	**0.952**	**506**

Lower MAE/RMSE and higher correlation are better. The Matched leaves column reports trajectory coverage—the number of ground-truth leaf identities matched to a predicted track (of 510 unique validation leaves); unmatched leaves are excluded from the per-leaf metrics, so coverage must be read alongside the error columns. For the adaptive method these downstream metrics and the matched-leaf count (506) are computed on the tracks from seed 42, not a cross-seed average (see Methods). Bold: best value.

Per-plant means across 37 validation plants; matched leaves is a count (of 510 unique validation leaves).

**Table 3:** Wilcoxon signed-rank test (two-sided): proposed method vs. LeafTrackNet on 37 paired validation plants.

Metric	Baseline mean	Ours mean	Δ	*p*-value
Emergence MAE	0.191	0.155	–0.035	0.505 (ns)
Lifespan MAE	0.505	0.391	–0.114	0.023^*^
LeafCount RMSE	0.461	0.367	–0.095	0.0005^***^
Area Corr	0.926	0.952	+0.026	0.098 (ns)

Δ = Ours mean − Baseline mean; Δ and the displayed means are computed from unrounded per-plant values, so the last displayed digit may differ by rounding. Significance symbols denote uncorrected two-sided Wilcoxon signed-rank *p*-values; Bonferroni-corrected interpretation is reported in the text.

#### 2.5.4 Difficulty-stratified CanolaTrack analysis

To test whether association improvements were restricted to easy validation plants or persisted across operating regimes, the CanolaTrack validation set was analyzed *post hoc* using GT-derived difficulty strata. No tracking outputs were retrained, regenerated, or threshold-tuned for this analysis. Plants were stratified at the plant-sequence level into tertiles (low/medium/high, *n* = 12*/*12*/*13 plants) for four difficulty factors, each computed per plant: leaf density (mean annotated leaves per frame), overlap (fraction of frames containing any leaf pair with IoU *>* 0.1), growth deformation (median |Δ*A/A*| over same-identity consecutive-frame leaf pairs), and spatial ambiguity (1 minus the mean nearest-neighbour centroid distance normalised by the image diagonal). Tertile boundaries are the 33rd and 67th percentiles of each factor across the 37 plants (leaf density 5.42/5.71 leaves per frame; overlap 0.36/0.39; growth deformation 0.21/0.22; spatial ambiguity 0.917/0.922). For each of the 12 resulting strata, total IDSW and mean association metrics were computed for Appearance-only, Fixed bio-constraints, LeafTrackNet, and Ours adaptive. Appearance-only served as the zero-reduction reference in the main difficulty figure; Centroid and IoU baselines were retained for the full tabular audit but not emphasised in the main figure.

#### 2.5.5 External association-only protocol

External evaluation was conducted as association-only testing on the Arabidopsis CVPPP Plant Phenotyping dataset (Minervini et al., 2016) and the KOMATSUNA dataset (Uchiyama et al., 2017). The Arabidopsis set comprises 12 plants (Ara2012: 4 plants, 13 daily frames; Ara2013-Canon: 8 plants, 17 daily frames) at approximately 24h intervals, which we partition into a 6-plant calibration split and a 6-plant test split by a fixed stack-ordered rule: the calibration split is Ara2012 stacks 01–02 and Ara2013Canon stacks 01–04, and the test split is Ara2012 stacks 03–04 and Ara2013-Canon stacks 05–08. All reported Arabidopsis metrics are computed over the 6 test plants (82 leaf identities and 1,004 leaf instances across 94 frames; the calibration split likewise contains 82 leaf identities across 94 frames). The exact plant-to-split assignment is listed in **Supplementary Table S6**. KOMATSUNA comprises 5 plants imaged over 10 days at six acquisitions per day (4h interval); we evaluate a daily-interval protocol using one frame per day (10 frames per plant; 34 leaf identities, 218 instances) and a full-sequence protocol using all 60 frames (37 leaf identities, 1,383 instances), under leave-one-plant-out calibration. These experiments used GT-derived boxes or masks as proposals. GT identity was never used as an online association input at test time: identity labels from the calibration plants were used only for target-domain calibration and retraining, whereas identities from the held-out test plants were used exclusively for evaluation. For Arabidopsis, per-leaf axis-aligned boxes were obtained by converting the dataset’s provided four-corner (rotated) leaf bounding-box annotations to axis-aligned boxes (the min/max of the four corners). For KOMATSUNA, per-leaf boxes were derived from the colour-coded label masks and only the RGB image was used (the depth channel of the RGB-D data was not used). For the external datasets, all appearance-based methods used the same ImageNet-pretrained MobileNetV3 crop embedding in a zero-shot manner: each proposal box is cropped, resized to 224 × 224, ImageNet-normalised, and embedded, and the resulting embedding is *L*_2_-normalised; the MobileNetV3 embedding encoder was not fine-tuned on external training or test plants. The tested association rules were Centroid, IoU, Appearance-only, Fixed bio-constraints, Ours-ZS, and Ours-Ext, each also in a target-domain calibrated form where indicated in **Table 4**. Here “ZS” (zero-shot) denotes zero-shot transfer of the CanolaTrack-trained Weight Predictor *parameters* only, not of the entire CanolaTrack tracker configuration. All external methods share the same post-Hungarian acceptance rule—a pair is accepted iff its assigned cost does not exceed *τ_c_*—but the underlying cost is method-specific: the composite biology-aware cost for the bio-aware and adaptive variants, the appearance cost for Appearance-only, the spatial cost for Centroid, and the IoU-based cost for IoU matching. This cost-based acceptance differs from the appearance-cosine acceptance rule used on CanolaTrack. The three calibrated forms change *different* components, and we state each explicitly. *Fixed bio-constraints (calibrated)* re-selects the four fixed blend weights on the calibration split, changing (*λ*_app_*,λ*_pos_*,λ*_area_*,λ*_life_) from the CanolaTrack default (1.0,0.3,0.2,0.1) to (0.0,0.7,0.1,0.0), with the rejection threshold left at *τ_c_*= 0.4. *Ours-ZS* applies the CanolaTrack-trained Weight Predictor with its network weights *unchanged* and changes only the scalar *τ_c_*(0.4 by default; 0.4→1.2 when calibrated on the split). *Ours-Ext* uses a Weight Predictor *retrained from scratch* on the 6 Arabidopsis calibration plants (three seeds 42/123/456; positive/negative pairs are consecutive-frame GT-matched/mismatched detections); its default form keeps *τ_c_*= 0.4 and its calibrated form uses *τ_c_*= 1.5 (the same retrained network in both). Thus “calibration” is not a single operation: Fixed-cal re-selects blend weights, Ours-ZS-cal re-selects *τ_c_*only, and Ours-Ext retrains the predictor (with an additional *τ_c_*selection). In this external association-only protocol *τ_c_*operates on each method’s assigned association cost (an assigned pair is accepted iff its cost does not exceed *τ_c_*); for the bio-aware and adaptive variants this is the composite cost, so calibrated values above 1 reflect that composite-cost scale, in contrast to the appearance-cosine threshold (*τ*_app_ = 0.4) used on CanolaTrack. All calibration and retraining use only the calibration split; the 6 test plants are never used for training or hyperparameter selection. KOMATSUNA results use the common threshold *τ_c_*= 0.4 for the default rows; older threshold-specific results are not used. The calibrated KOMATSUNA operating points (**Figure 4B**) use leave-one-plant-out calibration. For each of the five held-out test plants, all calibration and retraining use only the remaining four plants: Fixed bio-constraints re-selects the blend weights; Ours-ZS re-selects τ_c_ by grid search; and Ours-Ext retrains the Weight Predictor from scratch on the four non-test plants with seeds 42, 123, and 456, with its calibrated τc also selected without using the held-out plant. The held-out plant is used exclusively for evaluation, and the per-fold results are averaged.

**Table 4:** External association-only evaluation with GT-derived proposals—a deployment *operating boundary*, not a detector-transfer test.

Dataset/protocol	Method	IDSW↓	IDF1 (%)↑	AssA (%)↑	GT-instance coverage (%)↑
Arabidopsis (GT-box)	Centroid	11.2	79.5	68.5	100
	Fixed bio-constraints (default)	36.8	61.5	57.1	100
	Ours-ZS (default)	54.2	48.8	50.9	100
	Appearance-only	76.3	38.8	31.9	100
	IoU	73.0	41.5	43.3	100
	Fixed bio-constraints (calibrated)	8.0	84.1	75.7	100
	Ours-ZS ( $\tau_{c}$-calibrated)	11.5	77.7	71.1	100
	Ours-Ext (retrained + $\tau_{c}$-cal.)	**6.2**	85.2	77.7	100
KOMATSUNA daily	Centroid	1.6	93.4	81.6	100
	Fixed bio-constraints	9.2	71.0	66.4	100
	Ours-ZS	11.6	67.1	63.8	100
	Ours-Ext	14.1	59.1	56.7	100
	Appearance-only	10.4	62.2	52.9	100
	IoU	18.6	56.7	54.7	100
KOMATSUNA 60-frame	Centroid	0.8	98.7	93.7	100
	Fixed bio-constraints	15.8	82.0	78.6	100
	Ours-ZS	21.4	74.8	72.3	100
	Ours-Ext	23.5	72.6	70.3	100
	Appearance-only	17.4	78.4	70.8	100
	IoU	30.8	77.8	72.4	100

Default bio-aware rows use *τ_c_*= 0.4 (Fixed: CanolaTrack blend weights; Ours-ZS: the unchanged CanolaTrack-trained predictor); the Arabidopsis calibrated rows re-fit one component per method (Fixed: weights; Ours-ZS: *τ_c_*; Ours-Ext: retrained predictor +*τ_c_*; see Methods). KOMATSUNA calibrated leave-one-out points, including Ours-Ext, are in **Figure 4B**. IDSW (lower is better) is the mean per test plant—unlike the CanolaTrack total in **Table 5**—with the adaptive variants additionally averaged over three seeds (hence non-integer). GT-instance coverage = TP*/*(TP+FN) at IoU≥0.5 reflects output coverage, not identity correctness.

Bold indicates the lowest (best) IDSW value in the Arabidopsis comparison.

**Figure 4:**
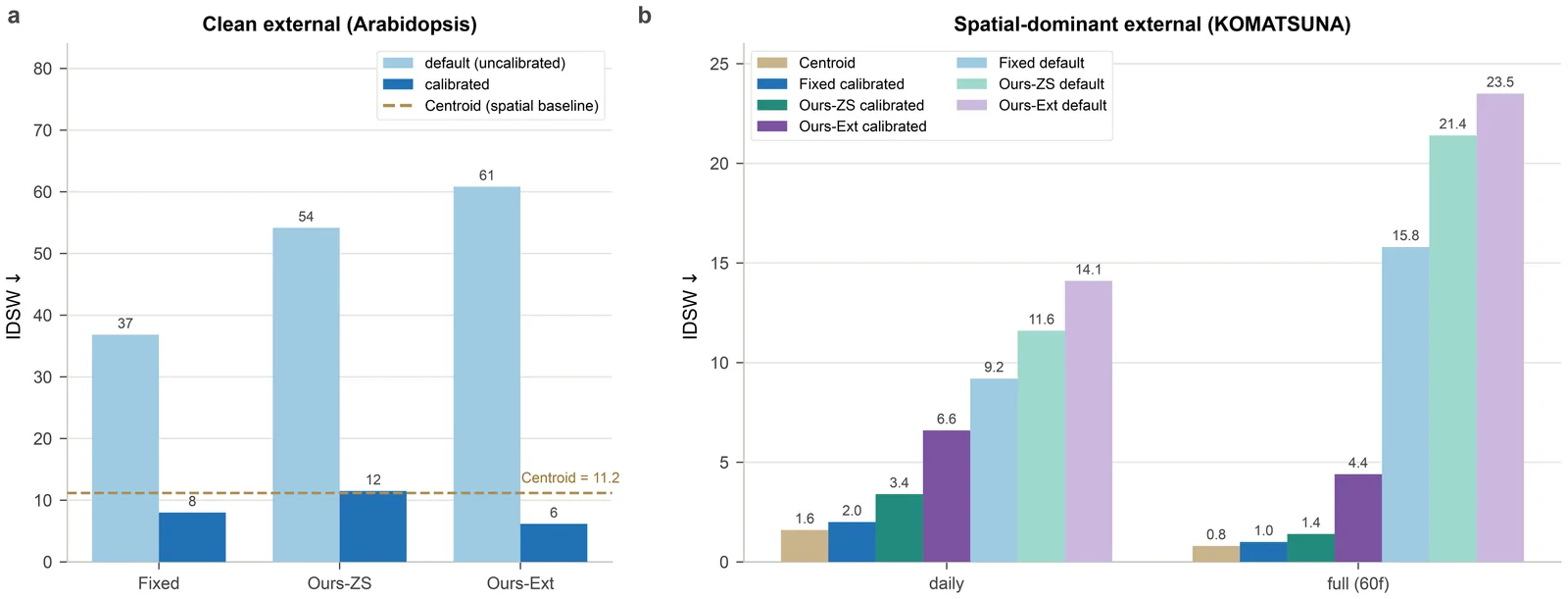
External operating envelope (association-only, GT-derived proposals; not a detector-transfer evaluation). **(A)** Arabidopsis (clean GT-box): light bars are the uncalibrated setting (*τ_c_*= 0.4), dark bars the target-domain calibrated form (Fixed: blend weights; Ours-ZS: *τ_c_*; Ours-Ext: retrained predictor +*τ_c_*; see Methods); the dashed line is the Centroid baseline (11.2 IDSW). **(B)** KOMATSUNA (well-separated leaves), daily and full-60-frame protocols, methods ordered by IDSW, with the retrained Ours-Ext shown alongside the zero-shot Ours-ZS. Both panels are interpreted in Section 4.2.

#### 2.5.6 Robustness perturbation protocol

Robustness was evaluated under the Arabidopsis association-only protocol using five random seeds (42,123,456,789,2026) and controlled proposal perturbations, each applied identically to all methods: bounding-box jitter (independent√ zero-mean Gaussian noise—box-centre standard deviation equal to the jitter level times $\sqrt{wh}$ and a multiplicative size factor $1+N(0,(0.5\;l{)}^{2})$ for level *ℓ*—at levels {0.05,0.10,0.20}), random missing detections (each detection dropped independently with probability {0.05,0.10,0.20,0.30}), injected false positives (adding round(*ℓ*×GT count) boxes per frame at uniformly random image positions with 20–80px sides and near-zero random appearance embeddings, at levels {0.05,0.10,0.20}), temporal sparsity (retaining every 1st, 2nd, 3rd, or 4th frame), and a combined moderate condition applying jitter 0.10, missing 0.10, and false positives 0.10 simultaneously. The analysis isolates the association stage under degraded proposal quality and should not be interpreted as full detector-transfer performance. Severe proposal degradation is treated as an engineering boundary: when detections are missing or poorly localised, association costs cannot recover leaf identities that are absent from the proposal set.

#### 2.5.7 Diagnostic interpretation protocol

Diagnostic analyses were used to interpret association behavior rather than to provide primary evidence. Cost-matrix and Weight Predictor diagnostics were examined to identify why appearance-only, centroid, fixed, and zero-shot adaptive methods behave differently across regimes. The external Weight-Predictor diagnostic is described as near-constant output or reduced context sensitivity under limited training-pair diversity, rather than as a formal mathematical collapse of the mapping.

## 3 Results

### 3.1 CanolaTrack benchmark and ablation

**Table 5** presents tracking performance across all evaluated methods, and **Figure 3** shows the per-plant paired improvement of the proposed method over the prior baseline and over our fixed-weight variant. To ensure comparability, the methods we evaluated used identical pre-computed detection proposals; appearance-based methods used the same MobileNetV3 embeddings from the official LeafTrackNet pipeline. This isolates the contribution of the association strategy from differences in detection quality, and, where appearance is used, from differences in feature extraction. The LeTra^∗^ row is not re-run here: it is reproduced from the CanolaTrack benchmark of Liu et al. (2025b), in which the original mask-based LeTra matching was adapted to bounding-box IoU (CanolaTrack provides only bounding-box annotations) and trained with its original implementation; that benchmark did not report IDSW.

**Table 5:** Tracking performance on CanolaTrack.

Domain	Method	HOTA↑	DetA↑	AssA↑	MOTA↑	IDF1↑	IDSW↓
Naive	Centroid+Hungarian	67.99	91.93	50.36	90.48	67.44	438
	IoU+Hungarian	77.50	92.19	65.24	91.16	78.05	395
General	DeepSORT (Wojke et al., 2017)	54.44	59.54	49.45	70.87	69.76	376
	ByteTrack (Zhang et al., 2022)	58.44	67.89	49.37	77.87	69.75	277
	BoT-SORT (Aharon et al., 2022)	58.44	67.89	49.37	77.87	69.75	278
Plant	LeTra^*^ (Jurado-Ruiz et al., 2024)	67.02 ± 0.04	82.03 ± 0.14	54.98 ± 0.16	82.09 ± 0.19	69.06 ± 0.10	n.r.
	LeafTrackNet^†^ (Liu et al., 2025b)	88.17	92.25	84.36	93.78	92.94	228
	**Ours**	**89.69 ± 0.10**	92.27 ± 0.03	**87.26 ± 0.19**	**95.39 ± 0.08**	**94.39 ± 0.09**	**126 ± 5**

Bold: best; underline: second best. IDSW is the total identity switches over the 37 validation plants; other metrics are percentages. All rows except LeTra^∗^ were evaluated under the unified shared-proposal protocol (Section 2.5); Ours is mean ± std over three seeds (42/123/456), LeafTrackNet^†^ is a single controlled reproduction, and the other baselines are single deterministic values. LeTra^∗^ reproduces the reported CanolaTrack benchmark of Liu et al. (2025b) (“n.r.” = IDSW not reported there). DetA is not highlighted, as it is fixed by the shared detector.

^†^LeafTrackNet is our single controlled reproduction under the shared official proposals and embeddings; the originally reported values (Liu et al., 2025b) (HOTA 88.03 ± 0.24, DetA 92.25 ± 0.03, AssA 84.07 ± 0.49, MOTA 93.64 ± 0.18, IDF1 92.90 ± 0.35) are consistent with it.

^∗^LeTra results are reproduced from the CanolaTrack benchmark of Liu et al. (2025b), in which the original mask-based LeTra matching was adapted to bounding-box IoU because CanolaTrack provides only bounding-box annotations; IDSW was not reported there.

General-purpose MOT trackers (DeepSORT, ByteTrack, BoT-SORT) achieve HOTA of only 54–58 under this protocol, more than 30 points below LeafTrackNet. This gap is consistent with the expectation that motion-based association frameworks designed for high-frame-rate pedestrian video are poorly matched to daily-interval plant imaging, where Kalman-predicted positions become unreliable across 24-hour intervals and IoU-based matching degrades as leaves grow and overlap between frames. The near-identical scores of the adapted ByteTrack and BoT-SORT configurations (IDSW 277 vs. 278) indicate no measurable net advantage of BoT-SORT under the static top-down CanolaTrack protocol.

Among plant-specific methods, the proposed approach achieves HOTA 89.69 ± 0.10, AssA 87.26 ± 0.19, IDF1 94.39 ± 0.09, and MOTA 95.39 ± 0.08. Relative to the strongest publicly available baseline on CanolaTrack, LeafTrackNet, identity switches are reduced by 44.7% (228 → 126 ± 5) and AssA increases by 2.90 points. DetA is essentially unchanged across the methods that share the detector’s proposals (LeafTrackNet 92.25, Ours 92.27 ± 0.03), confirming that the improvement originates in the biology-aware association stage rather than in detection. The small standard deviation across three independent training runs (± 0.10 HOTA, ± 5 IDSW) confirms that Weight Predictor training is stable and the results are not artefacts of a particular random initialisation. These results establish CanolaTrack as the primary operating regime where the adaptive association strategy is strongest.

#### 3.1.1 Fixed bio-constraints and adaptive weighting

**Table 6** reports the incremental contribution of each association cue under fixed weights (also shown graphically in **Supplementary Figure S2**). The Appearance-only baseline uses only *C*_app_. Adding spatial and area-growth constraints substantially reduces IDSW from 214 to 147. Adding the lifecycle plausibility cue does not further reduce IDSW under the adjacent-frame CanolaTrack protocol, and its aggregate contribution to the association cost is negligible (mean cost fraction ≈ 0): because track age is reset after each successful match, the age-gated lifecycle term is rarely active when most leaves are matched every frame. A direct sweep of the lifecycle weight *λ*_life_ (**Supplementary Table S4**) confirms this—removing the term entirely (*λ*_life_ = 0) changes aggregate performance negligibly (ΔHOTA −0.04, no change in IDSW).

**Table 6:** Ablation on association cues under fixed weights (single deterministic run).

App.	Pos.	Area	Life	HOTA↑	AssA↑	IDF1↑	IDSW↓
✓				88.58	85.17	93.37	214
✓	✓			88.85	85.64	93.43	163
✓	✓	✓		89.00	85.89	93.70	147
✓	✓	✓	✓	89.04	85.98	93.76	147

Checkmarks indicate active terms. Appearance is an association cue; position, area, and lifecycle are biological/geometric cues.

We nonetheless retain it as a principled prior for the leaf disappearance and reappearance challenge—the regime it is designed to address—and report its near-zero measured effect on CanolaTrack transparently rather than pruning it; its small positive effect on HOTA, AssA, and IDF1 is not presented as a primary contribution. We use the full fixed-weight configuration as the Fixed bio-constraints baseline.

**Table 7** compares the principal CanolaTrack association variants. The full fixed-weight bio-constraint configuration substantially reduces IDSW relative to Appearance-only (214 to 147) and LeafTrackNet (228 to 147); this reduction over LeafTrackNet is statistically robust in the per-plant paired analysis (median per-plant Δ = 2 IDSW, two-sided Wilcoxon *p* = 1.3 × 10^−4^, *n* = 37). The adaptive Weight Predictor provides the additional improvement needed in the CanolaTrack regime, reducing IDSW to 126 ± 5 and increasing AssA to 87.26 ± 0.19; this further adaptive-over-fixed reduction is smaller and only nominally significant (*p* = 0.049; see Section 4.1).

**Table 7:** CanolaTrack ablation summary.

Method	HOTA↑	AssA↑	IDF1↑	IDSW↓
LeafTrackNet (Liu et al., 2025b)	88.17	84.36	92.94	228
Appearance-only	88.58	85.17	93.37	214
Fixed bio-constraints	89.04	85.98	93.76	147
**Ours adaptive**	**89**.**69** ± **0**.**10**	**87**.**26** ± **0**.**19**	**94**.**39** ± **0**.**09**	**126** ± **5**

LeafTrackNet, Appearance-only, and Fixed bio-constraints all use the same detection proposals and MobileNetV3 embeddings; Appearance-only applies only the appearance cost within our tracker, whereas LeafTrackNet here is the published memory-based association procedure run under the same shared proposals and embeddings (a controlled association reproduction, not a re-training of the detector). Deterministic single-run configurations (Appearance-only, Fixed bio-constraints) and our single controlled LeafTrackNet reproduction are reported as single values, whereas Ours adaptive, which involves stochastic training, is reported as mean ± std over three seeds. Bold: best value.

### 3.2 Difficulty-stratified operating-regime analysis

**Figure 5** summarizes CanolaTrack performance across 12 GT-derived difficulty strata. Ours adaptive reduces IDSW in every stratum relative to Appearance-only and LeafTrackNet, with an average reduction of 40.0% versus Appearance-only and 45.0% versus LeafTrackNet. Fixed bio-constraints also provide a strong non-adaptive baseline, reducing IDSW by 30.1% on average versus Appearance-only. Adaptive weighting adds a further 14.0% average reduction over Fixed bio-constraints.

**Figure 5:**
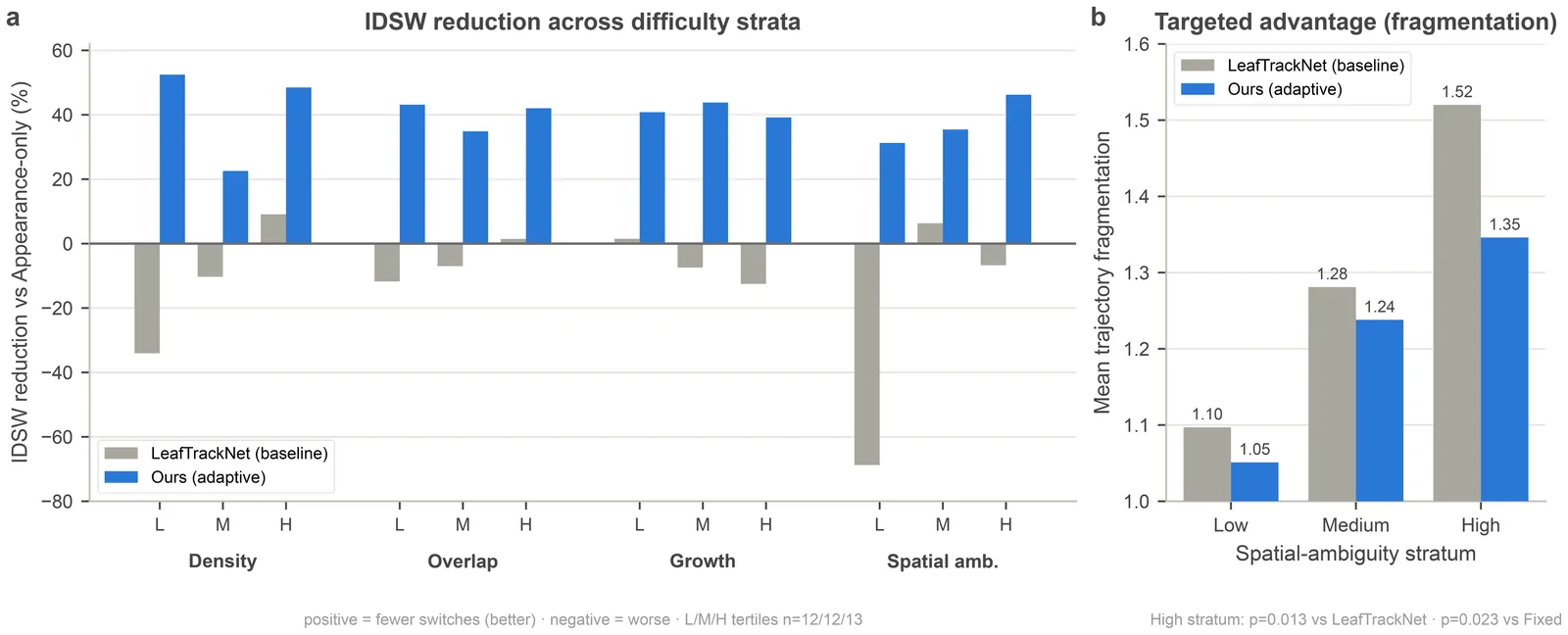
Difficulty-stratified operating-regime analysis on CanolaTrack. **(A)** IDSW reduction versus Appearance-only across four difficulty factors (density, overlap, growth deformation, spatial ambiguity), each in low/medium/high tertiles (*n* = 12*/*12*/*13); reduction = (IDSW_App_ − IDSW_method_)*/*IDSW_App_, positive = fewer switches. Ours reduces IDSW in every stratum; the prior LeafTrackNet baseline is uneven and worse than Appearance-only in several strata. **(B)** Mean trajectory fragmentation by spatial-ambiguity stratum (Ours vs LeafTrackNet *p* = 0.013; Ours vs Fixed *p* = 0.023). Fragmentation = distinct predicted IDs per ground-truth leaf (1 ideal); identity purity = fraction of a track’s detections matching its majority ground-truth identity (1 pure). Effect sizes and overall reliability statistics are in Section 4.1.

The gains are most directly visible in high-ambiguity strata. In the high-density stratum, IDSW decreases from 99 for Appearance-only and 90 for LeafTrackNet to 61 for Fixed bio-constraints and 51 for Ours adaptive. In the high-overlap stratum, the corresponding values are 69, 68, 44, and 40. Under high growth deformation, Ours adaptive reduces IDSW to 48.7 compared with 80 for Appearance-only, 60 for Fixed bio-constraints, and 90 for LeafTrackNet. In the high-spatial-ambiguity stratum, Ours adaptive reaches 64.

IDSW compared with 119, 82, and 127 for Appearance-only, Fixed bio-constraints, and LeafTrackNet, respectively. These results support stable CanolaTrack operating-regime gains, but they should not be read as a monotonic increase in benefit with every individual difficulty factor.

### 3.3 External association-only operating-envelope analysis

**Table 4** reports external GT-box association-only results on Arabidopsis and KOMATSUNA. These datasets represent cleaner, smaller, low-displacement regimes than CanolaTrack, and the ranking of association strategies changes accordingly. Among the methods without target-domain calibration, Centroid matching is strongest on both datasets, showing that simple spatial assignment can be sufficient when inter-frame displacement is minimal and proposals are clean; in this uncalibrated setting Fixed bio-constraints are the strongest bio-aware variant, whereas Ours-ZS does not support a claim of strong zero-shot adaptive-weight transfer. With the target-domain calibrated/adapted variants defined in Section 2.5 (blend-weight re-selection for Fixed, *τ_c_*recalibration for Ours-ZS, and predictor retraining for Ours-Ext), however, the biology-aware variants improve markedly and Ours-Ext attains the lowest Arabidopsis IDSW (Section 3.3, **Table 4**).

On Arabidopsis, Centroid obtains 11.2 IDSW and 79.5% IDF1. Fixed bio-constraints reduce IDSW from 76.3 for Appearance-only to 36.8, a 51.7% reduction, but remain below Centroid. Ours-ZS improves over Appearance-only in IDSW but underperforms Fixed bio-constraints, indicating domain bias in the CanolaTrack-trained Weight Predictor. On KOMATSUNA, Centroid remains strongest for both daily and full 60-frame protocols. Under the common *τ_c_*= 0.4 threshold (i.e. without target-domain calibration), Fixed bio-constraints are the strongest bio-aware variant in the external protocol. Ours-ZS does not consistently outperform Fixed bio-constraints, indicating limited and threshold-sensitive zero-shot transfer rather than robust cross-species adaptive generalisation.

### 3.4 Robustness under proposal perturbations

**Table 8** summarize Arabidopsis perturbation robustness. The clean condition repeats the *uncalibrated* external Arabidopsis comparison (default CanolaTrack weights, no target-domain calibration): among these methods Centroid is strongest in this small-displacement regime, and Fixed bio-constraints are the strongest bio-aware variant. With target-domain calibration and retraining where applicable (Section 2.5) the biology-aware variants recover and Ours-Ext attains the lowest IDSW on Arabidopsis (**Table 4**; **Figure 4A**). Under combined moderate perturbation, Centroid remains numerically strongest (27.5 IDSW, 51.0% IDF1), while Fixed bio-constraints approach Centroid in IDF1 (48.0%), substantially higher than Appearance-only (34.5%), Ours-ZS (35.3%), and IoU (24.6%). These results support Fixed bio-constraints as a reliable fallback under external proposal perturbations, not as an absolute winner under every severe noise condition.

**Table 8:** Arabidopsis robustness summary for clean and combined moderate proposal conditions.

Condition	Method	IDSW↓	IDF1 (%)↑	AssA (%)↑	GT-instance coverage (%)↑
Clean	Centroid	11.2	79.5	68.5	100.0
	Fixed bio-constraints	36.8	61.5	57.1	100.0
	Ours-ZS	54.2	48.8	50.9	100.0
	Appearance-only	76.3	38.8	31.9	100.0
	IoU	73.0	41.5	43.3	100.0
Combined moderate	Centroid	27.5	51.0	42.5	87.6
	Fixed bio-constraints	41.2	48.0	45.7	87.6
	Ours-ZS	61.7	35.3	38.6	87.6
	Appearance-only	72.0	34.5	28.1	87.6
	IoU	94.5	24.6	28.1	87.6

Values are averaged over five perturbation seeds where applicable. As in **Table 4**, IDSW is the mean number of identity switches per test plant (not a total). GT-instance coverage is defined as in **Table 4** (per-frame IoU≥0.5 matching, TP*/*(TP+FN)); it drops below 100% only when perturbations remove or displace detections.

### 3.5 Diagnostic interpretation

The external results are consistent with a regime-dependent interpretation of the association costs. Appearance-only matching lacks biological/geometric plausibility checks and is less robust when crops are visually similar. Centroid matching performs strongly on clean Arabidopsis and KOMATSUNA sequences because candidate displacement is small relative to the spatial threshold. Fixed bio-constraints retain this spatial and growth information without relying on an adaptive Weight Predictor trained in another domain. Ours-ZS may reject plausible external associations when the shifted geometry induces overly strong spatial weighting from the CanolaTrack-trained predictor, while its unsupervised near-constant lifecycle output can add a further age-gated penalty for coasting tracks. Diagnostics of externally trained predictors are therefore described as near-constant output or reduced context sensitivity under limited association-pair diversity, rather than a formal mathematical collapse of the weighting function.

On CanolaTrack, the learned per-pair weights depart systematically from the fixed defaults. Averaged over validation pairs, the adaptive predictor raises the position weight from 0.30 to 0.70 ± 0.32, the area weight from 0.20 to 0.40 ± 0.12, and the appearance weight from 1.00 to 1.16 ± 0.09. The lifecycle weight sits at a near-constant ≈1.0 across pairs; we read this as an unsupervised near-constant output rather than learned lifecycle behavior, because (Section 2.4) the lifecycle output receives no direct loss or gradient signal from the lifecycle term during training—its cost is identically zero on the gap-free training pairs—so its output value is not informative about lifecycle dynamics. An inference-only ablation on the three trained seeds (**Supplementary Table S5**) supports this reading: forcing the age feature to zero at inference has a negligible aggregate effect and leaves mean IDSW unchanged (IDSW 125.7→125.7), consistent with the feature never being trained at non-zero values, while forcing the predicted *λ*_life_ to zero increases IDSW only slightly (125.7→129.0)—the near-constant lifecycle weight acts as a mild constant inference-time prior, not a learned context-dependent behavior. The empirical adaptive gain is therefore attributable to the position and area weights and to the geometry-conditioned modulation of the appearance weight: among the supervised outputs, the position weight shows both the largest relative increase and the highest across-pair variance, consistent with spatial plausibility being upweighted and adaptively modulated where appearance is least discriminative. Correspondingly, the mean share of the total association cost carried by appearance falls from 75% under fixed weights to 65% under adaptive weighting, with the position share rising from 22% to 32%. We report these aggregate distributions as the evidence for adaptive behavior; statements about specific leaf types (small, mature, or transiently missing leaves) are treated as interpretation rather than directly measured claims.

### 3.6 Downstream phenotyping accuracy

**Table 2** reports downstream phenotyping metrics derived from tracking output for LeafTrackNet, the Fixed bio-constraints configuration, and the proposed adaptive method. **Table 3** reports Wilcoxon signed-rank test results from pairwise comparisons between Ours adaptive and LeafTrackNet across the 37 validation plants; the corresponding per-plant paired comparisons for all four downstream metrics are shown in **Supplementary Figure S1**. These results are retained as supporting evidence, not as the central claim of the study.

Ours adaptive is numerically best on all four downstream metrics, but the statistical support is limited. Leaf count RMSE decreases from 0.461 to 0.367 (*p* = 0.0005) and remains significant after Bonferroni correction over four metrics. Lifespan MAE decreases from 0.505 to 0.391 (*p* = 0.023), which is a nominal uncorrected improvement only. Emergence day MAE and area trajectory correlation do not reach statistical significance. The Fixed bio-constraints configuration improves IDSW on CanolaTrack, but it matches fewer leaves overall (465 matched leaves versus 506 for Ours adaptive and 504 for LeafTrackNet; **Table 2**). This lower trajectory coverage is consistent with more conservative effective association behavior, which may reject or fragment borderline matches, and with its weaker emergence, lifespan, and area-trajectory metrics despite its competitive leaf-count RMSE. Because the rejection rate was not measured directly, we treat this as a plausible explanation rather than a demonstrated mechanism. It nonetheless indicates that reducing identity switches alone is insufficient if an association strategy also reduces track continuity and trait coverage.

### 3.7 Inference time

**Table 9** reports per-frame association time measured on an NVIDIA RTX 4090. The proposed full adaptive method adds 0.165ms over the LeafTrackNet baseline (0.035ms), resulting in a total association time of 0.200ms per frame. In the context of daily-interval plant phenotyping, where images are not processed in real time and the total sequence length per plant is at most 31 frames, this overhead is entirely negligible. The additional cost relative to Fixed bio-constraints (0.050ms) arises from the MLP forward pass of Φ*_θ_*, which involves 2,788 trainable parameters.

**Table 9:** Per-frame association inference time on an NVIDIA RTX 4090.

Configuration	Time (ms/frame)
LeafTrackNet baseline	0.035
+ Fixed bio-constraints	0.050
+ Adaptive Weight Predictor (Ours)	0.200
Overhead (Ours vs. baseline)	0.165

## 4 Discussion

### 4.1 Why adaptive weighting works on CanolaTrack

The CanolaTrack regime combines daily growth, persistent overlap, changing leaf shape, and spatial ambiguity. In this setting, appearance similarity alone is less robust because overlapping leaves can produce visually similar crops, and appearance changes across 24-hour intervals can weaken the embedding signal. The fixed ablation shows that spatial continuity and area-growth plausibility already remove many implausible associations. Adaptive weighting is intended to add value because the usefulness of each cue can change with leaf context: small or newly visible leaves need not benefit from the same area constraint as larger mature leaves, and spatial plausibility may matter more when appearance is ambiguous. The learned per-pair weight and cost distributions (Section 3.5) are consistent with this account and bear on why Ours adaptive provides its largest gains in the intended CanolaTrack operating regime, but we present them as diagnostic evidence rather than as proof of a specific per-leaf mechanism.

The difficulty-stratified analysis strengthens this interpretation. Ours adaptive reduces IDSW across all 12 CanolaTrack difficulty strata relative to Appearance-only and LeafTrackNet. Against the prior LeafTrackNet baseline the per-plant reduction is large and highly significant (median Δ = +2.0 IDSW, 95% bootstrap CI [0.67,3.0], *p* = 1.6 × 10^−5^; **Figure 3A**). Its advantage *over Fixed bio-constraints*, however, is targeted rather than uniform: the per-plant paired test finds the adaptive-over-fixed IDSW reduction nominally significant by the paired Wilcoxon test (*p* = 0.049), with a median difference of zero and a bootstrap interval including zero, indicating a small and targeted effect, and the trajectory-reliability gain over Fixed is significant only in the high-spatial-ambiguity stratum (*p* = 0.023). Overall, Ours nonetheless improves trajectory reliability over LeafTrackNet (mean fragmentation 1.306 → 1.215, *p* = 0.002; identity purity 0.950 → 0.960, *p* = 0.028; two-sided paired Wilcoxon, *n* = 37). These results show that the benchmark improvement is not driven only by easy plants, while remaining honest that the learned increment over fixed constraints is concentrated where appearance and uniform weights are least informative. The analysis does not imply that the adaptive gain increases monotonically with every difficulty factor; rather, it shows a stable benefit over the prior baseline and a targeted benefit over fixed constraints.

### 4.2 Deployment boundary across external regimes

The Arabidopsis and KOMATSUNA experiments clarify the deployment boundary of adaptive weighting. Both external protocols use clean GT-derived proposals and small low-displacement association problems. Under those conditions, centroid matching is often sufficient because the correct detection is usually the nearest candidate. Fixed bio-constraints remain useful because they combine spatial and growth plausibility without relying on a CanolaTrack-trained Weight Predictor. Ours-ZS, by contrast, transfers adaptive weights learned from CanolaTrack into a different feature distribution, which can lead to over-rejection of plausible external associations.

A natural question is whether this weaker external adaptive performance reflects the low-displacement regime itself or simply the small size of these datasets limiting what an adaptive predictor can learn. The Ours-Ext results separate the two. On Arabidopsis, a Weight Predictor retrained from scratch on only six small calibration plants attains the lowest IDSW and beats Centroid (**Table 4**), so a small calibration set alone does not preclude a useful adaptive model. On KOMATSUNA—cleaner still, with well-separated leaves and minimal inter-frame displacement—the same retraining does not help: Ours-Ext stays well behind Centroid and Fixed bio-constraints and is no better than the zero-shot Ours-ZS (daily 14.1*/*6.6 vs 11.6*/*3.4 IDSW at default/calibrated *τ_c_*; 60-frame 23.5*/*4.4 vs 21.4*/*1.4). We read this as primarily a *regime* effect: when leaves are well separated and displacement is small, nearest-centroid assignment is already near-optimal and little association ambiguity remains for adaptive weighting to resolve, so retraining cannot add value; the very small, clean KOMATSUNA calibration folds additionally provide little training signal, consistent with Ours-Ext there matching rather than improving on zero-shot transfer. Adaptive weighting thus helps in proportion to the association difficulty of the regime—most on dense, ambiguous CanolaTrack, moderately on Arabidopsis, and not on the well-separated KOMATSUNA sequences.

This does not contradict the CanolaTrack result; it identifies the operating envelope. Dense daily canopy sequences with substantial overlap and spatial ambiguity are the appropriate target for adaptive weighting. Small clean low-displacement GT-box regimes may require only Centroid. Under domain shift, or when there is insufficient external association-pair diversity to train a stable Weight Predictor, Fixed bio-constraints are the more reliable cross-domain fallback.

### 4.3 Robustness and diagnostics

The robustness experiments support the same practical recommendation. Fixed bio-constraints are not the absolute winner under every perturbation, but they remain substantially stronger than Appearance-only and Ours-ZS under external Arabidopsis proposal perturbations. Under combined moderate noise, Fixed bio-constraints approach Centroid in IDF1 while retaining better association accuracy than the appearance-only and IoU baselines. This makes Fixed bio-constraints a useful fallback when proposal quality or domain alignment is uncertain.

The diagnostic analyses also caution against over-interpreting adaptive weights outside their training regime. The external Weight Predictor behavior is best described as near-constant output or reduced context sensitivity under limited training-pair diversity, rather than as a formal mathematical collapse of the mapping. Similarly, diagnostic case figures should be interpreted as cost-matrix evidence for association behavior rather than as exhaustive proof of every failure mode.

### 4.4 Implications for downstream phenotyping

The downstream phenotyping analysis remains useful but should be interpreted conservatively. Reducing IDSW can improve trajectory-derived traits because identity switches alter the time series assigned to individual leaves. In the 37-plant validation analysis, leaf count RMSE is the only metric that remains significant after Bonferroni correction. Lifespan MAE shows a nominal uncorrected improvement, while emergence day MAE and area trajectory correlation do not reach statistical significance. Therefore, the supported claim is that improved association can benefit selected downstream metrics, not that all phenotyping metrics significantly improve.

### 4.5 Limitations and future work

Several limitations constrain the conclusions. First, the external Arabidopsis and KOMATSUNA experiments are association-only GT-box protocols, not full detector-transfer evaluations. They identify association behavior under clean proposals, but they do not measure how a complete detector plus tracker would transfer across species or imaging systems. A full end-to-end cross-species evaluation— training or transferring a leaf detector per target species and comparing complete detector-plus-tracker pipelines, including LeafTrackNet, under a common protocol—is an important direction for future work that would complement the association-level analysis presented here. Second, those external datasets are low-displacement regimes and are not representative of all field or dense-canopy conditions.

Third, adaptive Weight Predictor training requires sufficient diversity in association-pair geometry. When external training data are limited, the predictor can show reduced context sensitivity, making Fixed bio-constraints a more reliable fallback. Relatedly, the present training pairs are all gap-free consecutive-frame transitions (track age 0, zero lifecycle cost), so the lifecycle branch of the predictor is not supervised; learning gap-aware, reappearance-specific weighting would require training pairs that explicitly span missing-detection gaps, which we leave to future work. Fourth, all association-stage methods depend on proposal quality: if leaves are missed, heavily mislocalized, or fragmented by the detector, the association module cannot recover identities that are absent from the proposal set. Finally, downstream phenotyping significance is limited to the supported metrics reported here; broader agronomic value remains to be tested in application-specific studies.

### 4.6 Conclusion

This study provides a biology-aware association framework and an operating-envelope analysis for leaf-level trait trajectory extraction. The framework combines one appearance cue with spatial, area-growth, and lifecycle plausibility cues, and can be deployed with either fixed bio-constraint weights or an adaptive Weight Predictor.

On CanolaTrack, adaptive weighting is most useful in the intended high-ambiguity regime, reducing IDSW from 228 to 126 ± 5 relative to LeafTrackNet and reducing IDSW across all 12 difficulty strata. External Arabidopsis and KOMATSUNA association-only experiments show a different boundary: in clean low-displacement GT-box regimes, centroid matching can be sufficient, and Fixed bio-constraints provide a more reliable cross-domain fallback than zero-shot adaptive-weight transfer.

The practical recommendation is therefore regime dependent. Use Ours adaptive for CanolaTrack-like dense daily canopy sequences with substantial overlap, growth deformation, and spatial ambiguity; use Centroid when clean low-displacement proposals make spatial nearest-neighbor assignment sufficient; and use Fixed bio-constraints when domain shift, limited Weight Predictor training diversity, or proposal perturbations make adaptive transfer unreliable.

## Data Availability

Publicly available third-party plant phenotyping datasets were analyzed in this study. The datasets include CanolaTrack, the Plant Phenotyping Datasets Arabidopsis time-series data, and the KOMATSUNA RGB-D dataset. The raw datasets are not redistributed with this submission and should be obtained from the original repositories cited in the article, subject to the original providers’ licenses and citation requirements. Source code, evaluation protocols, and analysis scripts for this study are available at https://github.com/awwbugbug/bioaware-leaf-tracking (Zhou and Zhang, 2025).
