# Supplementary material for: Biology-aware association for identity-consistent leaf tracking in top-down plant phenotyping
Source: Front Plant Sci. 2026 Oct 14;17:1881413. doi: 10.3389/fpls.2026.1881413 (PMC13713739; doi:10.3389/fpls.2026.1881413)
Supplement: Supplementary file 1 [file DataSheet1.pdf]

Supplementary Material

1 SUPPLEMENTARY FIGURES

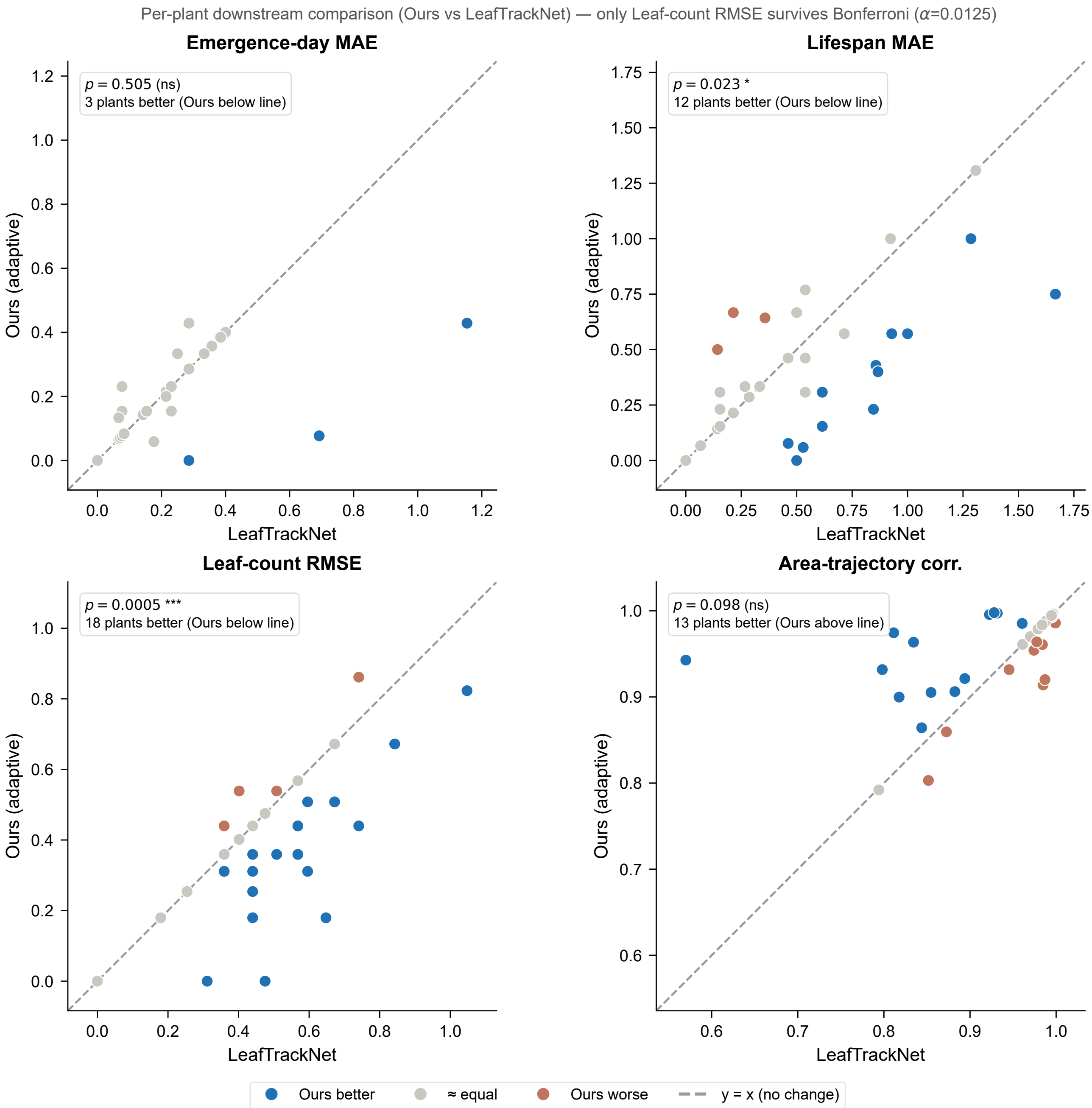

Figure S1. Per-plant downstream phenotyping comparison (Ours adaptive vs. LeafTrackNet) across the 37 CanolaTrack validation plants. Each point is one plant, plotting the Ours value (y) against the LeafTrackNet value (x); the dashed line is $y = x$ (no change between methods). For the three error metrics (emergence-day MAE, lifespan MAE, leaf-count RMSE) points below the line indicate that Ours is better; for area-trajectory correlation, points above the line indicate that Ours is better. Blue = Ours better, grey = approximately equal, red = Ours worse. Two-sided Wilcoxon signed-rank p-values are shown per panel; after Bonferroni correction over the four metrics ($\alpha = 0.0125$) only leaf-count RMSE remains significant.

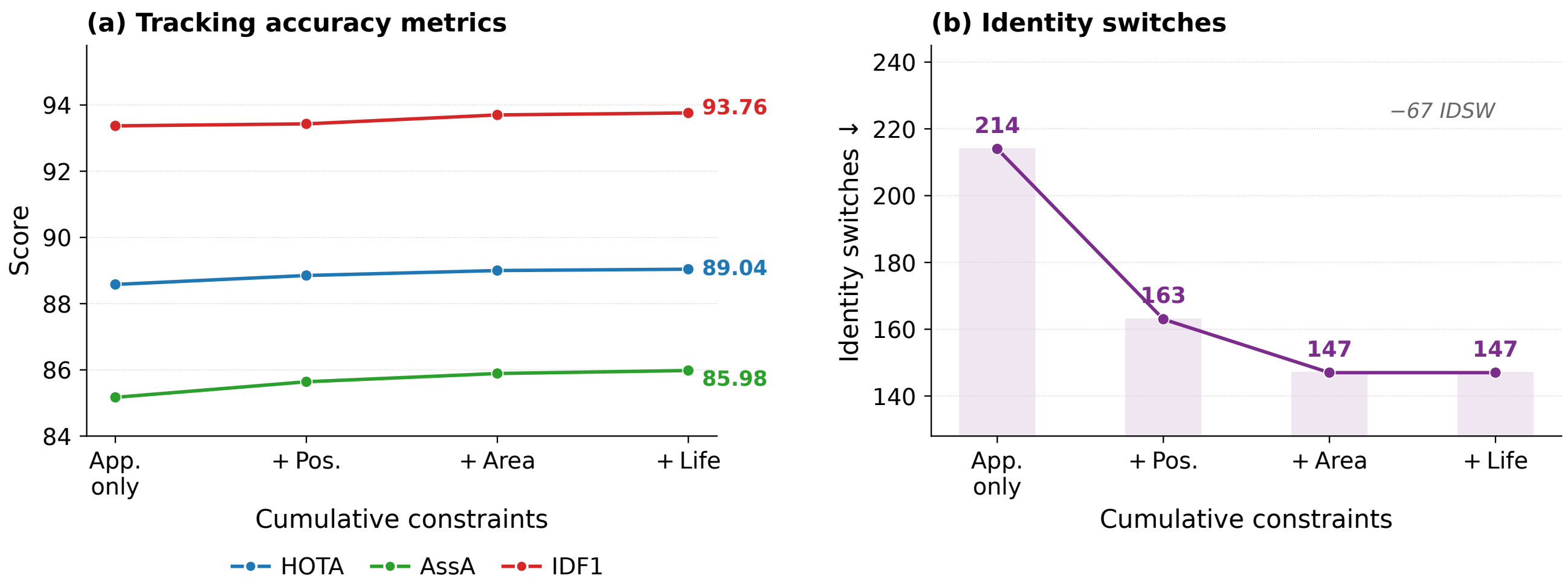

Figure S2. Cumulative ablation of association cues under fixed weights on CanolaTrack (single deterministic run). Beginning from appearance-only matching, the spatial-continuity and area-growth cues are added incrementally, followed by the lifecycle cue. This figure provides a graphical visualization of the cumulative ablation reported numerically in Table 3 of the main text.

2 SUPPLEMENTARY TABLES

Hyperparameter	Value	HOTA	AssA	DetA	IDF1	MOTA	IDSW
**Default**	**(all defaults)**	**89.04**	**85.98**	**92.28**	**93.76**	**95.06**	**147**
$d_{\max}$ (norm. scale)	0.15	88.95	85.80	92.29	93.46	95.00	151
	0.20	88.91	85.72	92.28	93.51	95.00	151
	0.30	88.47	84.95	92.22	93.21	94.84	161
	0.35	88.49	84.99	92.22	93.26	94.84	161
τ_A (area-shrink tol.)	0.20	88.85	85.62	92.28	93.55	94.98	152
	0.40	88.99	85.87	92.29	93.60	94.95	154
τ_a (max age)	3	89.13	86.17	92.27	93.71	95.11	144
	7	88.98	85.85	92.29	93.63	95.04	148
τ_{app} (app. cosine thresh.)	0.30	88.98	85.86	92.28	93.79	95.12	143
	0.50	88.89	85.67	92.30	93.46	94.84	161
λ_{pos}	0.2	88.78	85.49	92.28	93.46	94.87	159
	0.4	88.69	85.31	92.28	93.30	95.01	150
λ_{area}	0.1	88.99	85.89	92.28	93.60	94.89	158
	0.3	89.05	86.01	92.28	93.77	95.06	147
s_A (area-penalty scale)	0.5	88.99	85.89	92.28	93.60	94.89	158
	1.5	89.05	86.01	92.28	93.77	95.06	147

Table S1. Hyperparameter sensitivity of the fixed-weight biology-aware tracker on CanolaTrack validation. Each constraint hyperparameter is varied one at a time about its default while all others are held fixed. Across all tested configurations HOTA remains within $[88.47,\ 89.13]$ (range 0.66) and IDSW within $[143,\ 161]$. The default sits on a broad near-optimal plateau (τ_a=3, τ_{app}=0.3 and λ_{area}=0.3 match or slightly improve on it), and the strongest single direction of change is loosening the spatial normalisation scale ($d_{\max}\uparrow$). Over the tested CanolaTrack operating range, changing the area-penalty scale s_A produced the same aggregate metrics as a corresponding proportional change in λ_{area} (both scale the area cost, which enters as $\lambda_{\text{area}}\cdot\min(\text{penalty}\cdot s_A, 1)$; e.g. s_A=0.5 matches λ_{area}=0.1 and s_A=1.5 matches λ_{area}=0.3), indicating substantial empirical redundancy between the two area-scaling parameters; they are not strictly identical when the capped area penalty becomes active. Performance is therefore not the result of narrowly tuned parameter values but is robust across a wide operating band.

WeightPredictor seed	HOTA	AssA	DetA	IDF1	MOTA	IDSW
42	89.63	87.12	92.29	94.34	95.37	127
123	89.84	87.53	92.29	94.53	95.50	119
456	89.60	87.12	92.22	94.31	95.30	132
**Mean $\pm$ SD**	**89.69 $\pm$ 0.10**	**87.26 $\pm$ 0.19**	**92.27 $\pm$ 0.03**	**94.39 $\pm$ 0.09**	**95.39 $\pm$ 0.08**	**126 $\pm$ 5**
Fixed weights (ref.)	89.04	85.98	92.28	93.76	95.06	147

Table S2. Run-to-run consistency of the adaptive tracker across three independently trained WeightPredictor seeds. All three seeds outperform the fixed-weight baseline on HOTA, AssA, IDF1, MOTA, and IDSW, while DetA remains effectively unchanged under the shared proposal protocol (seed 456 is 92.22 vs. 92.28). The HOTA standard deviation is only ± 0.10, so the adaptive improvement is a reproducible, systematic gain rather than the effect of a single favourable initialisation. These three per-seed runs are the individual results aggregated in the Ours row of Table 2 of the main text (mean HOTA 89.69 ± 0.10, IDSW 126 ± 5); the fixed-weight reference row likewise matches the main-text value exactly (HOTA 89.04, IDSW 147).

WeightPredictor input	HOTA	AssA	DetA	IDF1	MOTA	IDSW
**Full (all five features)**	**89.63**	**87.12**	**92.29**	**94.34**	**95.37**	**127**
$-$ f_0 area ratio A_j/A_i	89.32	86.54	92.26	94.06	95.14	142
$-$ f_1 relative area change	89.63	87.12	92.29	94.29	95.36	128
$-$ f_2 normalised displacement	89.64	87.17	92.25	94.28	95.22	137
$-$ f_3 track age	89.60	87.06	92.29	94.31	95.37	127
$-$ f_4 fractional sequence position t/T	89.58	87.02	92.29	94.24	95.34	129

Table S3. Leave-one-out ablation of the five WeightPredictor input features. Each feature is masked identically at training and inference and the predictor is retrained (seed 42, otherwise identical protocol). The full five-feature model ties for the lowest IDSW (127, matched only by removing the inactive track-age feature); removing any single feature does not improve it. The area-ratio cue f_0 is dominant (removing it costs $+15$ IDSW and -0.31 HOTA) and normalised displacement f_2 is second ($+10$ IDSW). Removing the track-age feature f_3 leaves IDSW unchanged (127); this is expected because f_3 is *inactive under the present training construction*—all training pairs are gap-free consecutive-frame transitions with age $= 0$, so f_3 is identically zero during training and the predictor never learns to use it (it is not evidence of general redundancy). The relative-area-change f_1 and fractional sequence-position f_4 features contribute marginally given the others. Overall the predictor degrades gracefully rather than depending on any one fragile cue.

λ_{life}	HOTA	AssA	DetA	IDF1	MOTA	IDSW
0.00 (no lifecycle constraint)	89.00	85.89	92.30	93.70	95.06	147
0.05	89.02	85.93	92.30	93.71	95.07	146
**0.10 (default)**	**89.04**	**85.98**	**92.28**	**93.76**	**95.06**	**147**
0.20	89.07	86.04	92.28	93.77	95.07	146

Table S4. Sensitivity to the lifecycle-constraint weight λ_{life} (fixed-weight tracker). The lifecycle term $c_{\text{life}}(i,j) = (\text{age}_i/\tau_a) \cdot \hat{d}(i,j)$ is an age-weighted re-association penalty that is active only for dormant tracks (age > 0), discouraging a long-unmatched track from being claimed by a distant detection. Removing it entirely (λ_{life}=0) changes aggregate performance negligibly (ΔHOTA -0.04, ΔIDSW 0), consistent with the lifecycle term having limited aggregate influence on this benchmark. The constraint is thus a principled biological safety prior that does not materially alter aggregate performance, rather than a driver of the aggregate metrics; the empirical strength of the framework rests on the adaptive weighting (Table S2) and the spatial and area association cues, which carry the largest fixed-constraint reductions (main-text Table 3 and Supplementary Figure S2).

Inference-time configuration	HOTA	AssA	DetA	IDF1	MOTA	IDSW
**Full adaptive**	**89.71 ± 0.09**	**87.29**	**92.27**	**94.41**	**95.39**	**125.7 ± 5.0**
Age feature f_3 forced to 0	89.73 ± 0.09	87.33	92.27	94.42	95.39	125.7 ± 4.2
Predicted λ_{life} forced to 0	89.68 ± 0.17	87.24	92.27	94.44	95.34	129.0 ± 4.1

Table S5. Inference-only ablation of the adaptive model (three trained WeightPredictor seeds 42/123/456; no retraining; values are means across the three seeds, with standard deviation shown for HOTA and IDSW). Forcing the age feature f_3 to 0 at inference has a negligible aggregate effect and leaves mean IDSW unchanged ($125.7 \rightarrow 125.7$; HOTA $89.71 \rightarrow 89.73$), consistent with the feature never being trained at non-zero values (the age is identically zero throughout training), so it is inactive under this construction rather than merely redundant. Forcing the predicted lifecycle weight λ_{life} to 0 slightly increases IDSW ($125.7 \rightarrow 129.0$, ΔHOTA -0.03): although the lifecycle branch is unsupervised during training, its near-constant output (≈ 1.0) applies a mild age-gated penalty at inference, where tracks can coast (age > 0), and this yields a small benefit (≈ 3 fewer identity switches). The lifecycle weight thus acts as a constant inference-time prior rather than a learned, context-dependent behaviour. All three conditions come from a single internally-consistent re-run, so the reported differences are exact; the full-adaptive row reproduces the main-text Ours result (Table 2, HOTA 89.69, IDSW 126) exactly for two of the three seeds, differing by one identity switch on seed 456 because the currently available seed-456 checkpoint differs slightly from the one evaluated for Table S2 (all other settings identical).

Split	Plant ID	CVPPP source	Frames	Leaf IDs	Instances
Calibration	Plant-001	Ara2012 / stack_01	13	21	214
	Plant-002	Ara2012 / stack_02	13	18	176
	Plant-005	Ara2013-Canon / stack_01	17	12	163
	Plant-006	Ara2013-Canon / stack_02	17	9	120
	Plant-007	Ara2013-Canon / stack_03	17	12	164
	Plant-008	Ara2013-Canon / stack_04	17	10	117
	**Calibration total**		**94**	**82**	**954**
Test	Plant-003	Ara2012 / stack_03	13	18	206
	Plant-004	Ara2012 / stack_04	13	20	236
	Plant-009	Ara2013-Canon / stack_05	17	11	149
	Plant-010	Ara2013-Canon / stack_06	17	8	116
	Plant-011	Ara2013-Canon / stack_07	17	12	135
	Plant-012	Ara2013-Canon / stack_08	17	13	162
	**Test total**		**94**	**82**	**1,004**

Table S6. Arabidopsis CVPPP calibration/test split used for the external association-only evaluation. The 12 plants are partitioned by a fixed stack-ordered rule: the calibration split takes Ara2012 stacks 01–02 and Ara2013-Canon stacks 01–04, and the test split takes Ara2012 stacks 03–04 and Ara2013-Canon stacks 05–08. Plant IDs are the internal MOT-format identifiers; the CVPPP source column gives the corresponding original stack so the split is fully reproducible. All reported Arabidopsis metrics are computed over the 6 test plants; the test totals (94 frames, 82 leaf identities, 1,004 instances) match the values quoted in the main text. Calibration/retraining and threshold selection use only the calibration split; the test plants are never used for any tuning.
